# Intricacies of Mass Transport during Electrocatalysis: A Journey through Iron Porphyrin-Catalyzed Oxygen Reduction

**DOI:** 10.1021/jacs.4c04989

**Published:** 2024-05-23

**Authors:** Adarsh Koovakattil Surendran, Aleksandr Y. Pereverzev, Jana Roithová

**Affiliations:** Department of Spectroscopy and Catalysis, Institute for Molecules and Materials, Radboud University, Heyendaalseweg 135, 6525 AJ Nijmegen, The Netherlands

## Abstract

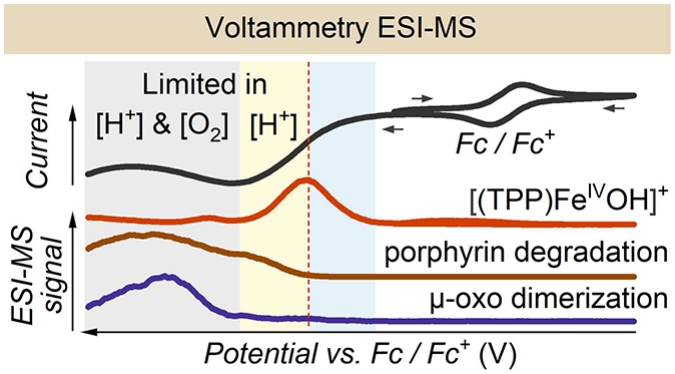

Electrochemical steps are increasingly attractive for green chemistry. Understanding reactions at the electrode–solution interface, governed by kinetics and mass transport, is crucial. Traditional insights into these mechanisms are limited, but our study bridges this gap through an integrated approach combining voltammetry, electrochemical impedance spectroscopy, and electrospray ionization mass spectrometry. This technique offers real-time monitoring of the chemical processes at the electrode–solution interface, tracking changes in intermediates and products during reactions. Applied to the electrochemical reduction of oxygen catalyzed by the iron(II) tetraphenyl porphyrin complex, it successfully reveals various reaction intermediates and degradation pathways under different kinetic regimes. Our findings illuminate complex electrocatalytic processes and propose new ways for studying reactions in alternating current and voltage-pulse electrosynthesis. This advancement enhances our capacity to optimize electrochemical reactions for more sustainable chemical processes.

## Introduction

Electrocatalytic processes hold immense promise in electrosynthesis and fuel cell technologies.^1,2^ Enhancing process efficiency is central to scaling these applications, a challenge intricately tied to understanding the reactions happening at the electrode interfaces.^3−5^ Traditional analytical methods often fail to capture the full spectrum of reaction dynamics, particularly those influenced by diffusion layers and concentration gradients that develop during electrolysis.^6,7^ Spectroelectrochemical methods, which couple electrochemistry with various spectroscopic techniques, have opened new windows to understanding complex electrochemical processes.^8−15^ Despite these advances, a more straightforward approach to comprehensively grasp the impact of dynamic electrode conditions on electrocatalytic efficiency remains a frontier to be conquered.^16^

Our investigation dives into this scientific frontier, focusing on heme-based molecular catalysts, especially iron tetraphenyl porphyrins, and their role in the electrochemical activation of dioxygen—a key process in the oxygen reduction reaction (ORR).^17,18^ This reaction progresses through a series of proton and electron transfer steps that have been well-studied in the past.^19−21^ Our research expands the scope to include the view of the evolving reaction conditions at the cathode on the known catalytic pathways and the less known or unknown catalyst decomposition pathways. Insights into catalyst degradation pathways during these processes have been notably elusive. Understanding these degradation mechanisms is vital for developing more robust and efficient electrocatalytic systems.^22−24^

We employ voltammetry and electrochemical impedance spectroscopy coupled with electrospray ionization mass spectrometry (VESI-MS and EIS-ESI-MS) to track electrochemical reaction intermediates in real time.^25^ These newly developed methods enable an unprecedented correlation between the recorded voltammograms and the detected intermediates, offering a unique perspective on how the developing diffusion layer and mass transport govern reactions at the electrode. Further, combining VESI-MS with cryogenic infrared photodissociation spectroscopy (IRPD) allows detailed characterization of the molecular structures of these intermediates. Through this comprehensive study, we aim to demonstrate the potential of VESI-MS for advancing the field of electrocatalysis/electrosynthesis and paving the way for developing more efficient and sustainable electrochemical technologies.

## Results

### Voltammetry Coupled with ESI-MS (VESI-MS)

**Figure 1:**
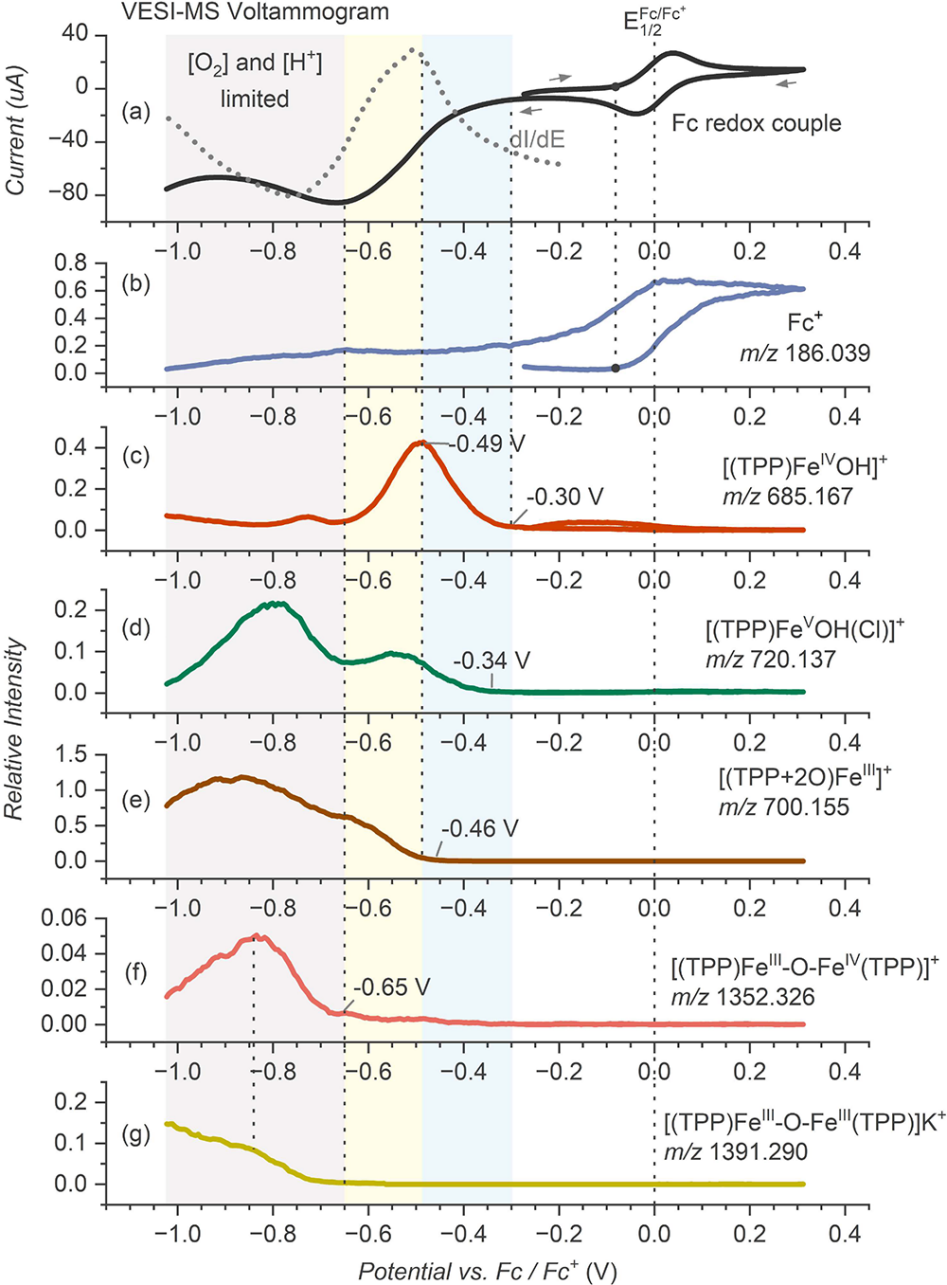
VESI-MS monitoring of electrocatalytic oxygen reduction reaction by the [(TPP)Fe^III^(Cl)] complex in the presence of 0.5 mM TFA. (a) The VESI-MS voltammogram trace (solid black) and its derivative (dotted gray). The extracted ion traces of (b) Fc^+^ ion (*m*/*z* 186.039), (c) [(TPP)Fe^IV^OH]^+^ (*m*/*z* 685.167), (d) [(TPP)Fe^V^OH(Cl)]^+^ (*m*/*z* 720.137), (e) [(TPP+2O)]Fe^III^]^+^ (*m*/*z* 700.155), (f) [(TPP)Fe^III^-O-Fe^IV^(TPP)]^+^ (*m*/*z* 1352.326), and (g) [(TPP)Fe^III^-O-Fe^III^(TPP)]K^+^ (*m*/*z* 1391.290). The ion abundances were normalized to the abundance of the parent [(TPP)Fe^III^(Cl)] complex (detected as [(TPP)Fe^III^(Cl)]K^+^) before applying the voltage (Figure S9). Experimental conditions: [(TPP)Fe^III^(Cl)] (0.1 mM) with TFA (0.5 mM) and electrolyte KPF_6_ (2 mM) in a DCM–MeCN mixture (1:3), measured at a scan rate of 5 mV s^–1^ under O_2_ pressure (0.12 bar).

We recorded the VESI-MS voltammogram (Figure 1a) of catalytic oxygen reduction by [(TPP)Fe^III^(Cl)] while simultaneously measuring the mass spectra of the intermediates formed on the working electrode during the reaction. The voltammogram was first scanned in the positive direction to calibrate the reference electrode potential by the ferrocene redox couple (Fc/Fc^+^) and further to the negative potential to record ORR. Calibration of the transfer time of the species from the electrode surface to the ESI-MS detection matches the onsets of the Fc^+^ signals in the voltammogram and the ESI-MS spectra (Figure 1b).

The onset of [(TPP)Fe^II^]-catalyzed ORR at −0.3 V correlates with detecting hypervalent hydroxo species [(TPP)Fe^IV^OH]^+^ (*m*/*z* 685.167) and [(TPP)Fe^V^OH(Cl)]^+^ (*m*/*z* 720.137) at −0.30 and −0.34 V, respectively (see the extracted ion traces in Figure 1c and d; for the mass spectra, see Figure S8). At about −0.46 V, a cation with *m*/*z* 700.155 corresponding to the molecular mass of the catalyst and two oxygen atoms [(TPP+2O)Fe^III^]^+^ was detected (Figure 1e; for the mass spectra, see Figure S8). Its appearance correlated with a decline of the [(TPP)Fe^IV^OH]^+^ abundance. Comparing the ion abundances with the voltammogram shows that the abundance of [(TPP)Fe^IV^OH]^+^ correlates with the derivative voltammogram (d*I*/d*E*), revealing the reaction rate (dotted gray curve in Figure 1a). The maximum of the derivative corresponds to the maximum rate of the O_2_ reduction to H_2_O, as indicated by detected [(TPP)Fe^IV^OH]^+^, which is the ultimate intermediate of the catalytic cycle. The catalysis slows down with the depleting proton concentration at the electrode. The maximum of the [(TPP)Fe^V^OH(Cl)]^+^ abundance is shifted by ∼−0.05 V. The onset of the [(TPP+2O)Fe^III^]^+^ detection correlates with the deceleration of the catalysis leading to [(TPP)Fe^IV^OH]^+^ and thus reveals a process switched on at low proton-concentration conditions.

At the mass-transport limit (−0.65 V), we start to see another chemical process yielding μ-oxo complexes [(TPP)Fe^III^-O-Fe^III^(TPP)] (detected as [(TPP)Fe^III^-O-Fe^III^(TPP)]K^+^, *m*/*z* 1391.290) and [(TPP)Fe^III^-O-Fe^IV^(TPP)]^+^ (*m*/*z* 1352.326) (Figure 1f and g; for the mass spectra, see Figure S8). The μ-oxo complexes are known to form in aerobic conditions in less acidic environments,^26^ correlating well with the mass-transport limitation of protons. Simultaneously, we detect a second rise of the signal intensities of [(TPP)Fe^V^OH(Cl)]^+^ and [(TPP+2O)]Fe^III^]^+^, suggesting another mechanistic pathway to their formation.

The mass-transport limitation mainly concerns the concentration of protons and oxygen molecules at the electrode during the reaction. To test the effect of the former, we studied the electroreduction at different concentrations of trifluoroacetic acid (TFA) (0.1, 0.25, and 0.5 mM, Figure S10). As expected, the catalytic current increased with the increased TFA concentration, which coincided with the enhanced abundance of [(TPP)Fe^IV^OH]^+^, [(TPP)Fe^V^OH(Cl)]^+^, and [(TPP+2O)]Fe^III^]^+^ formed during the catalysis (Figure S10c–e). The signal behavior at the derivative peak stayed qualitatively the same (Figure S11). Hence, at about −0.5 V, the signal of [(TPP)Fe^IV^OH]^+^ started to decline at the expense of the [(TPP+2O)]Fe^III^]^+^ signal. The formation of the μ-oxo complexes at the potentials below −0.65 V is slightly suppressed, as expected, with increasing acid concentration. Interestingly, the suppression of the [(TPP)Fe^III^-O-Fe^IV^(TPP)]^+^ abundance seems to correlate with a relative increase of the second rise of the [(TPP+2O)]Fe^III^]^+^ signal.

### Electrochemical Impedance Spectroscopy Coupled with ESI-MS

**Figure 2:**
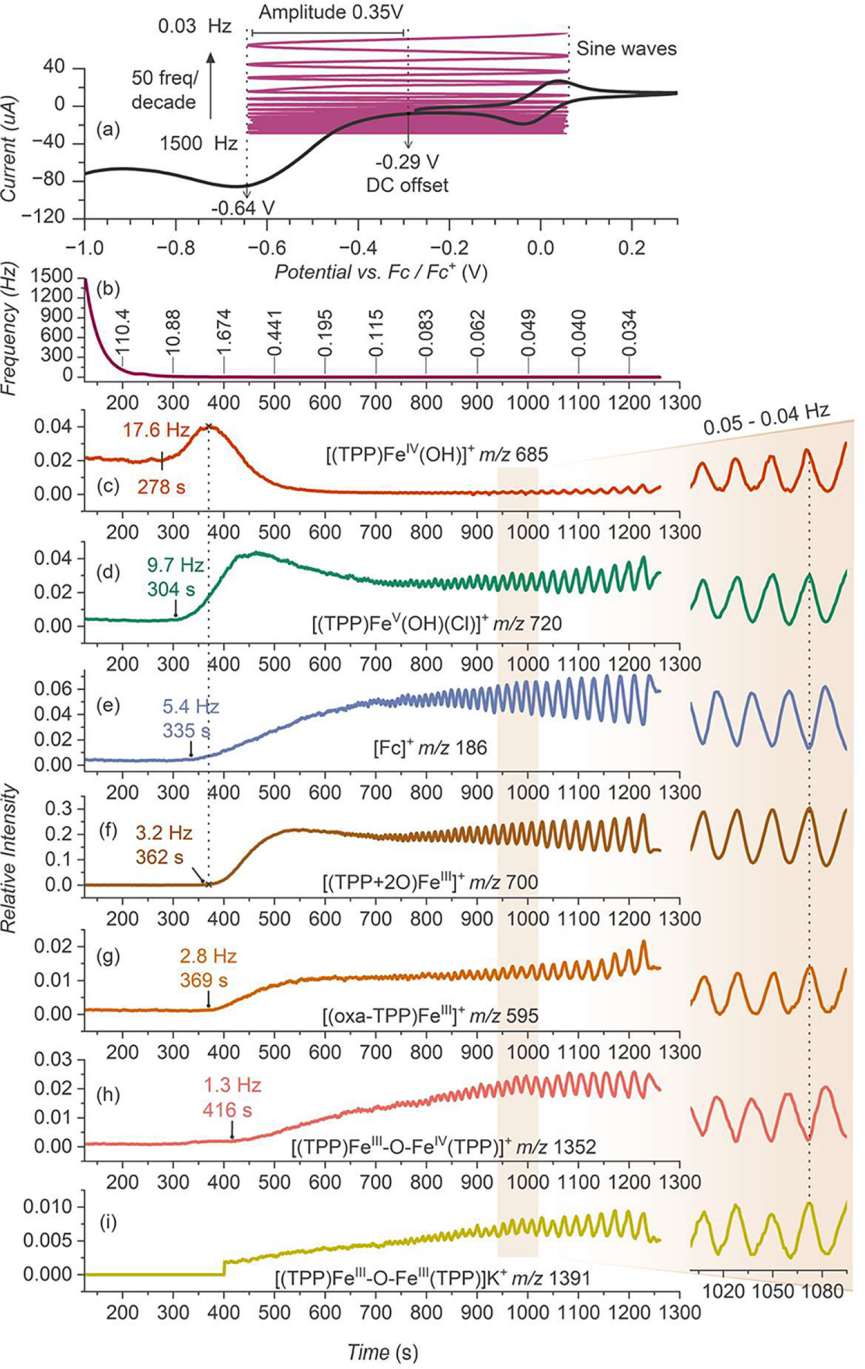
Electrochemical impedance spectroscopy coupled with electrospray ionization mass spectrometry (EIS-ESI-MS) for the analysis of oxygen reduction reaction by [(TPP)Fe^III^(Cl)]. (a) The voltammogram shows the DC offset and the AC perturbation applied for the analysis. (b) The applied scanning frequencies of the AC perturbation. (c–i) The extracted ion traces from the ESI-MS spectra: (c) [(TPP)Fe^IV^OH]^+^ (*m*/*z* 685.167), (d) [(TPP)Fe^V^OH(Cl)]^+^ (*m*/*z* 720.137), (e) ferrocenium (*m*/*z* 186.039), (f) [(TPP+2O)]Fe^III^]^+^ (*m*/*z* 700.155), (g) [(oxa-TPP)]Fe^III^]^+^ (*m*/*z* 595.121), (h) [(TPP)Fe^III^-O-Fe^IV^(TPP)]^+^ (*m*/*z* 1352.326), and (i) [(TPP)Fe^III^-O-Fe^III^(TPP)]K^+^ (*m*/*z* 1391.290). The frequencies of the AC perturbation are scanned at 50 frequencies per decade. The ion abundances were normalized to the abundance of the parent [(TPP)Fe^III^(Cl)] complexes (detected as [(TPP)Fe^III^(Cl)]K^+^) before applying the voltage. On the right: enlarged signals in the 990–1090 s range. Experimental conditions: [(TPP)Fe^III^(Cl)] (0.1 mM), TFA (0.5 mM), KPF_6_ (2 mM) in a DCM–MeCN mixture (1:3), under an O_2_ atmosphere (0.12 bar).

Electrochemical impedance spectroscopy (EIS) provides kinetic information on electrochemical reactions by differentiating electrochemical reactions at various time scales.^27,28^ EIS operates by perturbing an electrochemical system at a steady state or equilibrium with a sinusoidal signal (current or voltage) and measuring the system’s response (voltage or current) across a broad frequency spectrum. Coupling EIS with ESI-MS adds real-time molecular-level information and can thus be a powerful tool for studying the kinetics and mechanisms of electrochemical reactions (Figure 2).

The EIS-ESI-MS analysis of the [(TPP)Fe]-catalyzed ORR was performed at the DC offset of −0.29 V and an alternating current (AC) sinusoidal perturbation with a 0.35 V amplitude, covering the voltage range 0.06 to −0.64 V (vs Fc/Fc^+^) (Figure 2a). Standard EIS measurements apply a small AC amplitude (10 to 20 mV) to ensure a linear relationship between the applied signal voltage and the measured system’s response (current).^27^ For this demonstration, we applied a large amplitude to increase the concentration of the formed intermediates and, thereby, the detection efficiency. The focus was detecting the intermediates/species formed at the electrode in response to the applied sinusoidal perturbation. The perturbation signal was scanned at 50 frequencies per decade, from 10^5^ Hz to 0.03 Hz (the 1500 to 0.03 Hz range is shown in Figure 2). The detected ion signals share a common profile, in which the signal rises at a certain time/frequency point. As the voltage oscillations go toward lower frequencies, the ion signals rise and then start to oscillate with a growing amplitude. The signal growth and the growing amplitude reflect an increasing concentration and concentration gradient at the electrode, resulting from the longer reaction times allowed by lower voltage frequencies.

The EIS-ESI-MS analysis confirms that the [(TPP)Fe^IV^OH]^+^ ions are the first intermediates detected with nonzero abundance already at the applied DC potential. The [(TPP)Fe^IV^OH]^+^ signal further rises at the voltage oscillation frequency of 17.6 Hz (278 s, Figure 2c). Slightly later, at 304 s, corresponding to 9.7 Hz, the signal [(TPP)Fe^V^OH(Cl)]^+^ rises (Figure 2d), followed by the ferrocenium signal at 5.4 Hz (335 s, Figure 2e; the ferrocenium signal appearance at a lower frequency is a consequence of the longer reduction pulse along with its high reversibility). Similar to the VESI-MS measurements, we observe a depletion of [(TPP)Fe^IV^OH]^+^ correlated with the formation of [(TPP+2O)]Fe^III^]^+^ at 362 s and 3.2 Hz (Figure 2f). Almost simultaneously, at 369 s/2.8 Hz, new ions with *m*/*z* 595.121 appeared (Figure 2g). These ions were not observed during the voltammetry experiments, which suggests that their formation requires a longer reaction time. Accordingly, we confirmed the accumulation of these ions during a 1 h chronoamperometry experiment (Figure S12). Finally, the mass-transport limit appears when the voltage period at the reduction phase (−0.29 to −0.64 V) is long enough for the reaction to reach the diffusion limit, and the reactions leading to the generation of μ-oxo complexes were detected at 416 s/1.3 Hz (Figure 2h and i).

The detection of the ions depends on the reaction rate at the electrode to generate a minimum detectable concentration. Hence, the observed frequency does not directly correspond to the electrochemical rate, but it does contain information about the rate. The rise and the shape of the detected ion abundance are related to the concentration of the species at the electrode (see also Figure S7). The [(TPP)Fe^IV^OH]^+^ ions appear at the highest frequency and disappear at a frequency when their formation becomes proton-limited, and other reactions prevail. This result suggests that the [(TPP)Fe^IV^OH]^+^ intermediates are formed in the fastest reaction and do not accumulate in solution. At very low frequencies, the [(TPP)Fe^IV^OH]^+^ intermediates can be detected again, and their abundance oscillates with the voltage. The [(TPP)Fe^IV^OH]^+^ abundance is closely tied to the proton availability; therefore, their reappearance at low voltage-oscillation frequencies suggests that the frequency dropped to the range compatible with the proton diffusion rate.

The EIS-ESI-MS traces of all other detected ions indicate that these ions can accumulate in the solution. Interestingly, the detected μ-oxo complexes are counter-correlated (see the [(TPP)Fe^III^-O-Fe^III^(TPP)]K^+^ and [(TPP)Fe^IV^-O-Fe^III^(TPP)]^+^ signals in detail on the right side), suggesting their connection by a reversible redox process. Hence, the [(TPP)Fe^IV^-O-Fe^III^(TPP)]^+^ complexes are formed dominantly by oxidation of the [(TPP)Fe^III^-O-Fe^III^(TPP)] complex during the EIS-ESI-MS experiment. On the contrary, the [(TPP)Fe^IV^-O-Fe^III^(TPP)]^+^ ions detected during the voltammetry experiments above have a different origin because only reduction reactions are possible.

### Spectroscopic Characterization of the Detected Intermediates

**Figure 3:**
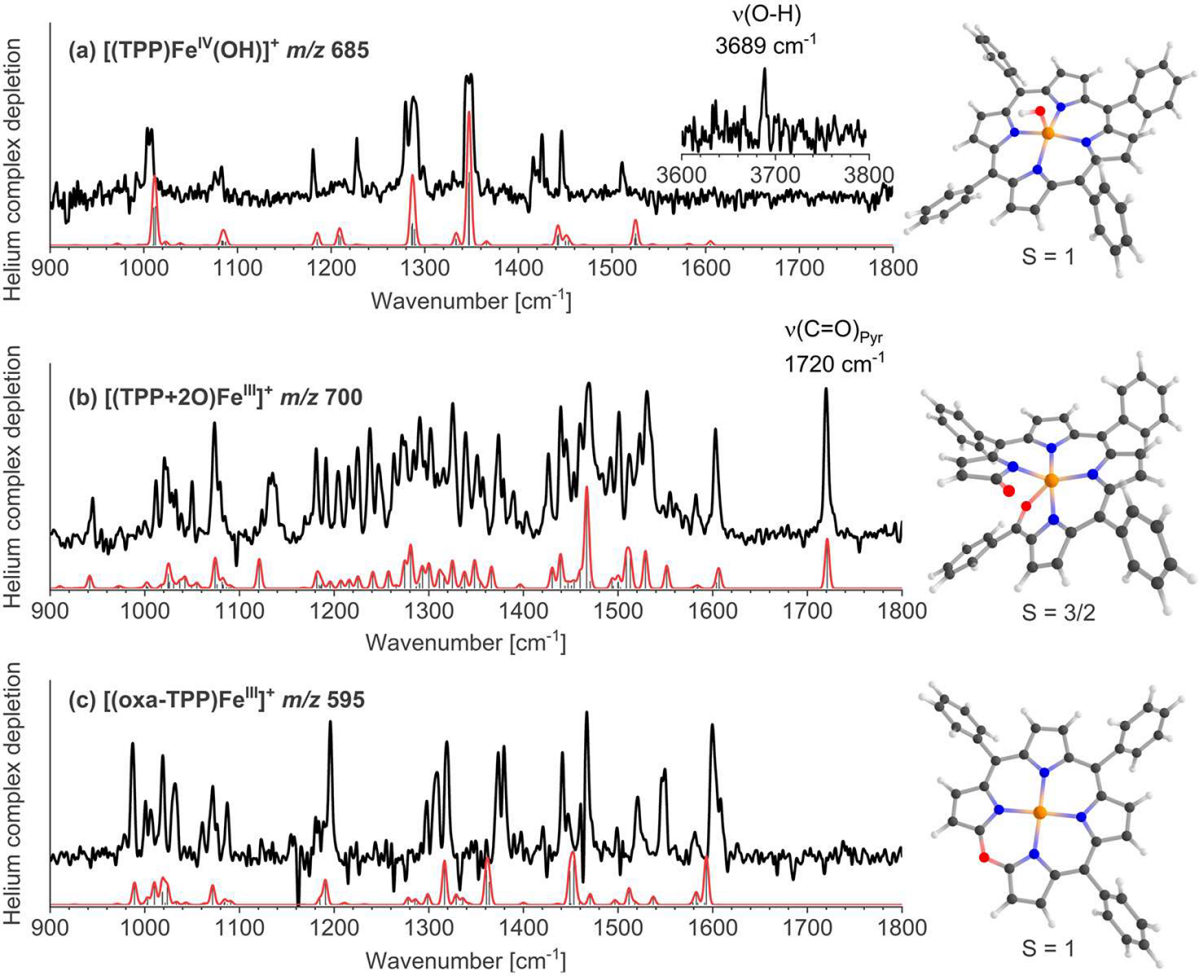
Helium tagging infrared photodissociation spectra of mass-selected (a) [(TPP)Fe^IV^OH]^+^ (*m*/*z* 685.167), (b) [(TPP+2O)]Fe^III^]^+^ (*m*/*z* 700.155), and (c) [(oxa-TPP)]Fe^III^]^+^ (*m*/*z* 595.121) with the DFT-calculated spectrum overlaid in red for the given structures.

Helium tagging infrared photodissociation spectroscopy is an advanced cryogenic mass spectrometric technique that provides IR spectra of mass-selected ions.^29−34^ We integrated the VESI-MS setup with IRPD spectroscopy, creating a powerful analytical method for assigning molecular structure to the detected intermediates during the electrochemistry experiments. We have measured the IRPD spectra of all detected monomeric complexes and assigned them based on the comparison with DFT calculations (Figure 3 and Figures S13–S17). The IRPD spectra of [(TPP)Fe^III^]^+^, [(TPP)Fe^IV^OH]^+^, and [(TPP)Fe^V^OH(Cl)]^+^ correspond to the expected ground-state structures of the iron complexes. The hydroxo and chloro ligands are in the axial positions, and the O–H stretching vibrations are at 3689 cm^–1^ for [(TPP)Fe^IV^OH]^+^ and 3636 cm^–1^ for [(TPP)Fe^V^OH(Cl)]^+^ (see Figure 3a and Figure S13).

The structure of the [(TPP+2O)]Fe^III^]^+^ complex could correspond to the iron(III)-superoxo complex. However, its fragmentation pattern does not show any elimination of O_2_. Instead, the complex eliminates a fragment corresponding to the benzoyl radical (Figure S18). The IRPD spectrum, showing a carbonyl band at 1720 cm^–1^, is significantly more complex than the spectra measured for the parent [(TPP)Fe^III^]^+^ and other intermediates, suggesting the degradation of the porphyrin ring. Accordingly, we could assign the experimental spectrum to the complex with a cleaved porphyrin ring at one of the pyrrole links (see Figure 3b). Finally, the ions with *m*/*z* 595.121 accumulating at longer reaction times have the same mass as the products of the benzoyl loss from the [(TPP+2O)]Fe^III^]^+^ complexes. The IRPD spectrum of these ions can be assigned to oxa-porphyrin complexes (Figure 3c). Hence, the IRPD spectra show that we detected intermediates and products of porphyrin degradation during the electrocatalytic ORR. This degradation pathway is analogous to the biochemical path of heme degradation via verdoheme (oxa-porphyrin) to biliverdin.^35,36^ The same pathway was also suggested for the stoichiometric reaction between iron porphyrin and H_2_O_2._^37,38^

## Discussion

**Figure 4:**
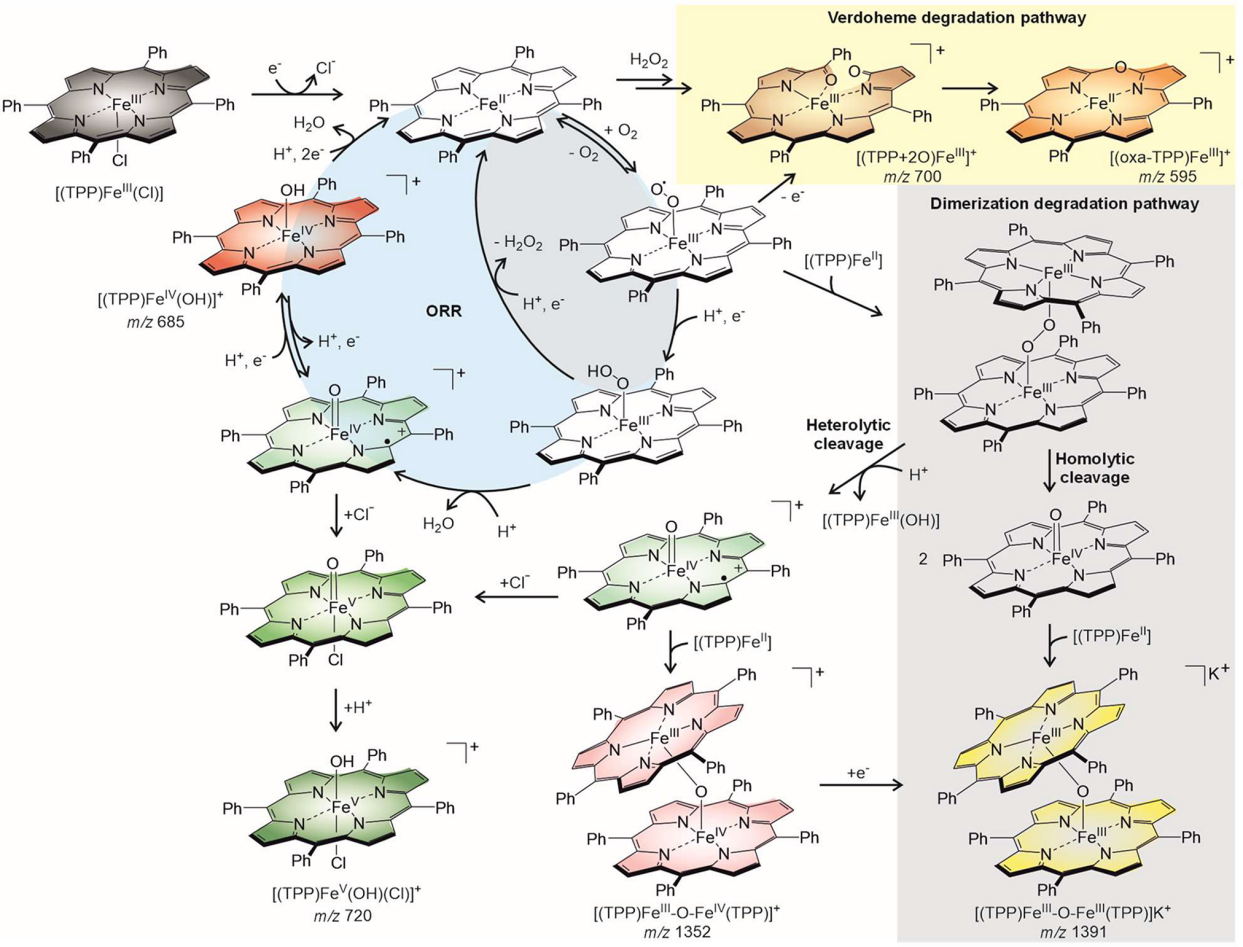
Electrocatalytic oxygen reduction reaction mechanism catalyzed by [(TPP)Fe^II^].

The VESI-MS and EIS-ESI-MS analyses unveil reaction and degradation pathways in electrochemical ORR catalyzed by the [(TPP)Fe^II^] complex. The catalytic cycle starts with reducing [(TPP)Fe^III^(Cl)] to the active form [(TPP)Fe^II^]. The [(TPP)Fe^II^] complex reacts with dioxygen, and in a series of proton and electron transfer reactions, O_2_ is transformed into two molecules of H_2_O. If the proton transfer steps do not limit the reaction, the reaction is fast (*k* > 17 Hz detected under our experimental settings). At the fastest time scale, we initially detect only the last intermediates in the catalytic cycle, [(TPP)Fe^IV^OH]^+^ (red in Figure 4), suggesting that all previous steps are faster when there are enough protons or the preceding intermediates are neutral. We detect neutral intermediates protonated or tagged by the potassium cation; however, the sensitivity is lower than that of naturally charged intermediates, and they can thus escape the detection. At a slightly longer time scale, we can also detect [(TPP)Fe^V^OH(Cl)]^+^, which is most likely the product of stabilization of the in-cycle [(TPP^•+^)Fe^IV^(O)]^+^ intermediates (the compound I analog) by attaching Cl^–^ and detected as protonated ions (Figure 4 in green).^39,40^ With sufficient proton concentrations, the [(TPP^•+^)Fe^IV^(O)]^+^ undergoes fast proton-coupled electron transfer to [(TPP)Fe^IV^OH]^+^. This reaction gets slower with decreasing proton concentration, which makes the Cl^–^ association competitive. Hence, the detected offset in the onset of [(TPP)Fe^V^OH(Cl)]^+^ in the VESI-MS experiments and the time delay in the EIS-ESI-MS experiment with respect to [(TPP)Fe^IV^OH]^+^ is caused by the interplay among these reactions and changing proton concentration. Protonation of the off-cycle [(TPP^•+^)Fe^IV^(O)(Cl)] intermediate likely occurs during the transfer time or electrospray ionization.

As the reaction starts to be proton-concentration limited, the abundance of [(TPP)Fe^IV^OH]^+^ decreases at the expense of [(TPP+2O)]Fe^III^]^+^ (see the verdoheme degradation pathway in Figure 4 and the yellow-highlighted panel in Figure 1). The proton transfer limitation leads to the accumulation of the [(TPP)Fe^III^(OO)] superoxide complex that most likely self-oxidizes to form the [(TPP+2O)]Fe^III^]^+^ intermediate and further degrades at a longer reaction time to the oxa-porphyrin (verdoheme) product. The self-oxidation pathway must be triggered by electron transfer. The accumulation of the [(TPP+2O)]Fe^III^]^+^ intermediate coincides with the accumulation of the [(TPP)Fe^V^OH(Cl)]^+^ intermediate. Hence, it is likely that electron transfer between these two species opens the verdoheme degradation pathway. Alternatively, one could suggest that proton limitation favors the H_2_O_2_ catalytic path,^41,42^ and H_2_O_2_ then reacts with the catalyst, resulting in degradation.^37,43^

At even more negative potentials (the gray-highlighted panel in Figure 1), we see the onset of the reaction pathways leading to the μ-oxo complexes. This reaction pathway is most likely triggered by the mass-transport limit of protons and oxygen molecules. The latter leads to the [(TPP)Fe^II^] accumulation at the electrode. The dimerization paths start with the reaction between the [(TPP)Fe^III^(OO)] superoxide complex and the [(TPP)Fe^II^] complex (see the gray panel in Figure 4).^26^ Protonation of the [(TPP)Fe^III^-O–O-Fe^III^(TPP)] adduct can trigger a heterolytic bond cleavage to generate [(TPP^•+^)Fe^IV^(O)]^+^ and [(TPP)Fe^III^OH]. This pathway explains the second rise of the [(TPP)Fe^V^OH(Cl)]^+^ and [(TPP+2O)]Fe^III^]^+^ abundances and the minor abundance of the [(TPP)Fe^IV^OH]. The [(TPP^•+^)Fe^IV^(O)]^+^ complex can alternatively react with another [(TPP)Fe^II^], forming the [(TPP)Fe^IV^-O-Fe^III^(TPP)] complex. The reaction sequence is finished by reducing this complex to the stable, unreactive [(TPP)Fe^III^-O-Fe^III^(TPP)] dimer.

The [(TPP)Fe^III^-O-Fe^III^(TPP)] complex can also be formed via the homolytic cleavage pathway of the initial [(TPP)Fe^III^-O-O-Fe^III^(TPP)] adduct. The cleavage yields two [(TPP)Fe^IV^(O)] complexes, which react with another [(TPP)Fe^II^] to form stable, unreactive [(TPP)Fe^III^-O-Fe^III^(TPP)]. These complexes accumulate in the solution, and we can see their accumulation and periodic oxidation during the EIS-ESI-MS experiments (Figures 2 and 3).

## Conclusion

We presented a new way of investigating the reaction processes at the electrodes during electrocatalysis. Hyphenating voltammetry or electrochemical impedance spectroscopy with electrospray ionization mass spectrometry opens an insight into the mechanisms and kinetics of the electrochemical reactions at the molecular frontier. The experiments provide direct feedback on evolving reaction conditions at the electrode–electrolyte interphase by detecting changes in the concentrations of reaction intermediates or products. We demonstrate this approach for electrochemical oxygen reduction catalyzed by the [(TPP)Fe^II^] complex. We detected and characterized reaction intermediates and catalyst degradation pathways operating at different kinetic regimes during electrocatalysis. The demonstrated molecular insight under dynamic electrochemical conditions opens possibilities to unravel reactions during AC and voltage-pulse electrosynthesis.^44,45^ It also potentially offers an efficient tool for studying reaction kinetics at the electrodes to find optimum conditions and for pairing electrochemical reactions during AC electrolysis.^46−48^

## Experimental Methods

A PalmSens USB-powered potentiostat (PalmSens4) was used for VESI-MS measurements, and the EIS measurements used a Metrohm potentiostat (PGSTAT204) installed with an FRA module. A digital electronic back pressure regulator (EL-PRESS P-702CV-21KR-RAD-11-K) obtained from Bronkhorst was used to monitor and maintain a constant headspace gas pressure. VESI-MS analysis was conducted with a Bruker trapped ion mobility time-of-flight (timsTOF) mass spectrometer with an electrospray ionization (ESI) source. In this study, the timsTOF was operated as a typical TOF mass spectrometer with the ion mobility separation turned off. The following ESI settings were used to transfer the ions: capillary voltage 4 kV, dry heater 200 °C, dry gas 2 L min^–1^, and nebulizer gas 0.5 bar. The detector (TOF) was calibrated for the masses before the measurements using a low-concentration tuning mix (ESI-L Part No. G1969-85000) from Agilent Technologies.

The VESI-MS setup is a single-compartment gastight voltammetric cell with a Pt pseudoreference electrode, a Pt mesh counter electrode, and a specially designed Toray carbon working electrode. The setup closely resembles a standard voltammetric cell arrangement. The critical premise of the experiment is the collection and transfer of the species generated at the working electrode surface/vicinity to the mass spectrometer. The best-performing solution is based on the design of a working electrode from two Toray carbon paper sheets with a silica capillary sandwiched between them. The capillary collects the in situ generated species from the Toray carbon surface and transfers them to the mass spectrometer by a flow induced by gas overpressure. The flow rate is controlled by varying the applied headspace gas pressure; a constant pressure is maintained during the measurement using a digital electronic back pressure regulator (Bronkhorst, EL-PRESS P-702CV-21KR-RAD-11-K) attached to the headspace of the cell. Polarization of the VESI-MS cell was controlled by the PalmSen4 (battery/USB-powered) potentiostat operated via Bluetooth in the floating mode. The complete details about developing and validating the VESI-MS method and further technical information can be found elsewhere.^25^

The VESI-MS method allows us to monitor the formation of charged species at the electrode surface during a voltammetric scan and compare the traces of the detected ions with the voltammogram as a function of the applied potential. The VESI-MS voltammograms were recorded at a 5 mV s^–1^ scan rate in a solution of acetonitrile–dichloromethane (MeCN–DCM) (3:1), containing [TPPFe^III^(Cl)] (100 uM), potassium hexafluorophosphate (KPF_6_) electrolyte (2 mM), ferrocene (Fc) internal standard (100 μM), and different concentrations of trifluoroacetic acid (100, 250, and 500 μM) under a constant oxygen overpressure (0.12 bar). The cell was filled with 2 mL of the solution for a single measurement; after every scan, the cell and electrodes were washed with solvent, and the cell was refilled with a fresh solution. The transfer time for the species from the electrode surface to the mass spectrometer was determined by measuring the delay in the appearance of the ferrocenium signal when an oxidation pulse (+0.1 V) was applied (typically 10 s, Figure S7). This approach led to perfect syncing of the Fc^+^ signal appearance in the voltammogram and the mass spectra (see more experimental details and results in the Supporting Information).

Ion spectroscopic measurements were performed in our home-built spectrometer ISORI (ion spectroscopy of organic reaction intermediates) using helium tagging.^29,30^ In a typical experiment, the intermediates/ions generated ([M]^+^) using the VESI-MS setup were mass-selected by a quadrupole mass filter and guided into a cold quadrupole ion trap (∼3 K) by a quadrupole bender and an octopole ion guide. The ions were trapped and thermalized in collisions with helium buffer gas. The cold ions formed helium-tagged complexes ([M(He)]^+^) that were used to monitor IR photon absorption. The trapped [M(He)]^+^ ions were irradiated by a Nd/YAG laser-pumped tunable OPO/OPA system (Laser Vision) that operated at a 10 Hz repetition rate. After the irradiation, the [M(He)]^+^ ions were extracted from the trap, mass-analyzed by a quadrupole, and detected with a Daly-type detector working in the counting mode. The photon absorption (ν_i_) was monitored as a depletion of the number of [M(He)]^+^ complexes. The counts of [M(He)]^+^ complexes were measured in alternating cycles (1 Hz) with (*N*(ν_i_)) and without (*N*_0_) the laser beam admitted to the ion trap. The infrared photodissociation (IRPD) spectra are derived as the attenuation 1 – *N*(ν_i_)/*N*_0_ plotted against the infrared wavenumber.

## Supporting Information Available

General information, VESI-MS method, synthesis of [(TPP)Fe^III^(Cl), helium tagging IRPD, and DFT (PDF)
