# Supplementary material for: Intricacies of Mass Transport during Electrocatalysis: A Journey through Iron Porphyrin-Catalyzed Oxygen Reduction
Source: J Am Chem Soc. 2024 May 23;146(22):15619–26. doi: 10.1021/jacs.4c04989 (PMC11157527; doi:10.1021/jacs.4c04989)
Supplement: Supplementary file 1 — ja4c04989_si_001.pdf [file ja4c04989_si_001.pdf]

Supplementary Information for

Intricacies of Mass Transport during Electrocatalysis: A Journey through Iron Porphyrin-Catalyzed Oxygen Reduction

Adarsh Koovakattil Surendran, Aleksandr Y. Pereverzev, and Jana Roithová

Department of Spectroscopy and Catalysis, Institute for Molecules and Materials, Radboud University, Nijmegen, The Netherlands

Corresponding author: j. roithova@science.ru.nl

Table of Contents

1. Materials and Methods

1.1 General Information…… 3

1.2 VESI-MS Method…… 3

1.3 Synthesis of H_2TPP…… 4

1.4 Synthesis of [(TPP)Fe^{III}(Cl)]…… 4

1.5 Helium Tagging IRPD Spectroscopy…… 4

1.6 DFT…… 5

2. Experimental Results

2.1. ^1H NMR Spectrum of H_2TPP…… 6

2.2. Mass Spectrum of H_2TPP……7

2.3. Mass Spectrum of [(TPP)Fe^{III}(Cl)]…… 8

2.4. Solution Resistance Measurement…… 9

2.5. IR-Drop Correction……10

2.6. Transfer Time Determination…… 11

2.7. Mass Spectra of the Intermediates Detected during VESI-MS…… 12

2.8. VESI-MS Analysis - All the Ions Detected…… 13

2.9. VESI-MS Analysis at Different Concentrations of TFA…… 14

2.10. VESI-MS Analysis at Different Concentrations of TFA - Correlation with the Derivatives... 15

2.11. Accumulation of *m/z* 595 during Chronoamperometry…… 16

2.12. IRPD Spectrum and DFT Optimized Structures of the Intermediates…… 17

2.13. Isomers of $[(TPP+2O)Fe]^+$ - Comparison with IRPD Spectrum…… 18

2.14. Isomers of $[(TPP)Fe^{V}OH(Cl)]^+$ - Comparison with IRPD Spectrum…… 20

2.15. Isomers of $[(TPP)Fe^{IV}OH]^+$ - Comparison with IRPD Spectrum…… 22

2.16. Isomers of $[(oxa\text{-}TPP)]Fe^{III}]^+$ - Comparison with IRPD Spectrum…… 23

2.17. Collision-Induced Dissociation Spectra (CID)…… 24

3. References

1. Materials and Methods

1.1. General Information

Chemicals purchased from commercial sources were used without further purification. Potassium hexafluorophosphate (99%), iron(II) chloride tetrahydrate ($FeCl_2.4H_2O$) (98%), sodium sulfate (Na_2SO_4) (99%), benzaldehyde (99%), propionic acid (99.5%) were obtained from Sigma Aldrich. Ferrocene (98%) and pyrrole (99%) were sourced from Acros Organics. Dichloromethane (DCM) (99.8%), dimethyl formamide (DMF) (99%), and methanol (99.8%) were obtained from Fisher Scientific and acetonitrile (MeCN) (HPLC grade) from VWR. Toray carbon paper (TGP-H-060) was obtained from Fuel cell earth. Platinum wire and platinum gauze (99.9%) were from Alfa Aesar. The fused silica capillary (ID 100 µm, OD 190 µm, part #: Z-FSS-100190) was purchased from Postnova.

A PalmSens USB-powered potentiostat (PalmSens4) was used for VESI-MS measurements, and the EIS measurements used a Metrohm potentiostat (PGSTAT204) installed with an FRA module. A digital electronic back pressure regulator (EL-PRESS P-702CV-21KR-RAD-11-K) obtained from Bronkhorst was used to monitor and maintain a constant headspace gas pressure. VESI-MS analysis was conducted with a Bruker trapped ion mobility time of flight (timsTOF) mass spectrometer with an electrospray ionization (ESI) source. In this study, the timsTOF was operated as a typical TOF mass spectrometer with the ion mobility separation turned off. The following ESI settings were used to transfer the ions: capillary voltage 4 kV, dry heater 200 ℃, dry gas 2 L/min, and nebulizer gas 0.5 bar. The detector (TOF) was calibrated for the masses before the measurements using a low-concentration tuning mix (ESI-L Part No: G1969-85000) from Agilent Technologies.

1.2. VESI-MS Method

The VESI-MS setup is a single-compartment gas-tight voltammetric cell with a Pt pseudo reference electrode, a Pt mesh counter electrode, and a specially designed Toray carbon working electrode. The setup resembles close to a standard voltammetric cell arrangement. The critical premise of the experiment is the collection and transfer of the species generated at the working electrode surface/vicinity to the mass spectrometer. The best-performing solution is based on the design of a working electrode from two toray carbon paper sheets with a silica capillary sandwiched between them. The capillary collects the in situ generated species from the toray carbon surface and transfers them to the mass spectrometer by a flow induced by gas overpressure. The flow rate is controlled by varying the applied headspace gas pressure; a constant pressure is maintained during the measurement using a digital electronic back pressure regulator (Bronkhorst, EL-PRESS P-702CV-21KR-RAD-11-K) attached to the headspace of the cell. Polarization of the VESI-MS cell was controlled by palmsens4 (battery/USB-powered, USB = universal serial bus) potentiostat operated via Bluetooth in the floating mode. The complete details about developing and validating the VESI-MS method and further technical information can be found elsewhere (*1*). Due to the absence of a frequency response analyzer in the floating USB/battery potentiostat, the electrochemical impedance (EIS) measurements were done using a separate potentiostat (Metrohm Autolab, PGSTAT204). The potentiostat used for VESI-MS measurements does not support IR compensation during measurement, so the applied potential was corrected for the IR drop after the measurement. The solution resistance was determined using electrochemical impedance spectroscopy (Figure S5), and a post-measurement correction of the IR drop was applied to the scanning voltages. Without IR compensation during the measurement, the applied scanning voltages may deviate from linearity (depending on the magnitude of the current flowing), resulting in scanning rate variability (shown in Figure S6). Analysis, especially a quantitative interpretation of the data, must consider the possible involvement of scan rate variability. Initially, we analyzed the data qualitatively; however, the scanning rate variability was also considered (see supplementary Figure S6b).

The VESI-MS method allows us to monitor the formation of charged species at the electrode surface during a voltammetric scan and compare the traces of the detected ions with the voltammogram as a function of the applied potential. The VESI-MS voltammograms were recorded at a 5 mV s^{-1} scan rate in a solution of acetonitrile/dichloromethane (MeCN/DCM) (3:1), containing [$TPPFe^{III}$(Cl)] (100 uM), potassium hexafluorophosphate (KPF_6) electrolyte (2 mM), ferrocene (Fc) internal standard (100 uM), and different concentrations of trifluoroacetic acid (100 uM, 250 uM and 500 uM) under a constant oxygen overpressure (0.12 bar). The cell was filled with 2 mL of the solution for a single measurement; after every scan, the cell and electrodes were washed with solvent, and the cell was refilled with a fresh solution. The electrolyte's choice and the concentration are based on their compatibility with mass spectrometry measurements. A higher concentration of the supporting electrolyte can cause ion suppression, electrospray discharge, solidification, and blocking the entrance of the mass spectrometer. The Fc/Fc^+ redox couple was used to calibrate the reference electrode potential. The transfer time for the species from the electrode surface to the mass spectrometer was determined by measuring the delay in the appearance of the ferrocenium signal when an oxidation pulse (+ 0.1 V) was applied (typically 10 seconds, Figure S7). This approach led to perfect syncing of the Fc^+ signal appearance in the voltammogram and the mass spectra.

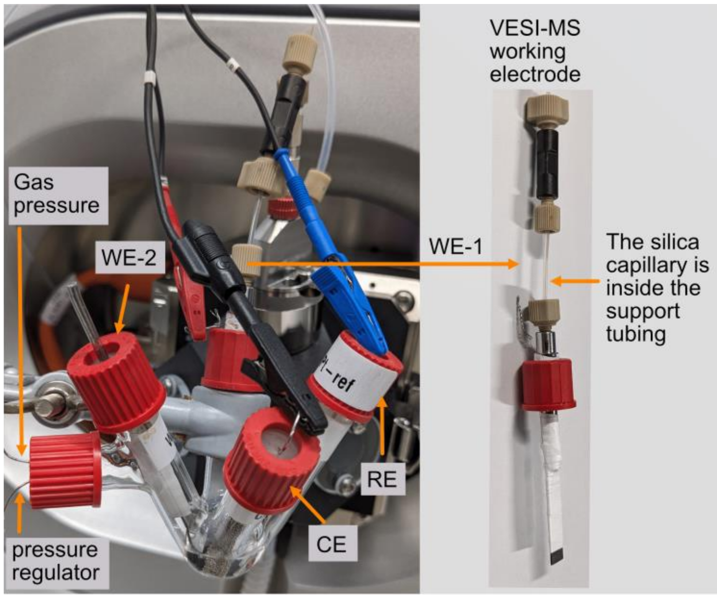

Figure S1: The VESI-MS cell coupled to the electrospray ionization (ESI) mass spectrometer. WE-1: Primary VESI-MS working electrode is connected to the mass spectrometer through a silica capillary, RE: Platinum pseudo reference electrode, CE: platinum mesh counter electrode, WE-2: a low surface area secondary working electrode (from the same Toray carbon paper of the WE-1) is used to obtain the reference/trial voltammograms and the identified electrochemical potential window of interest is scanned on the primary VESI-MS working electrode at a slow scan rate 5 mV s^{-1}.

1.3. Synthesis of 5,10,15,20-tetrakis(phenyl)-21*H*,23*H*-porphyrin (H_2TPP)

H_2TPP was synthesized following a general procedure (*2*). 10 mL (10.4g, 98.4 mmol) of benzaldehyde was mixed with 500 mL of propionic acid under continuous stirring. Further, pyrrole 7 mL (6.8g, 100 mmol) was added to the reaction mixture and stirred under reflux for 4 hours. The reaction mixture was cooled to room temperature and kept in the fridge overnight for precipitation of porphyrin. The reaction mixture was filtered, washed with methanol, and dissolved in dichloromethane (DCM). The

H_2TPP dissolved in DCM was then purified using silica gel column chromatography to obtain a yield of 27 % (4.08 g).

1H NMR (fig. S1) (400 MHz, Chloroform-d, TMS) δ (ppm) 8.84 (s, 8H), 8.26 – 8.18 (m, 8H, Ph-CH), 7.83 – 7.70 (m, 12H, Ph-CH), -2.77 (s, 2H). ESI-MS (fig. S2) : $[M+H]^+$, *m/z* 615.26

1.4. Synthesis of Iron (III) 5,10,15,20-tetrakis (phenyl)porphyrin [(TPP)Fe^{III}(Cl)]

H_2TPP (250 mg, 407 μmol) and $FeCl_2.4H_2O$ (450 mg, 2.26 mmol) were dissolved in 100 mL dimethylformamide (DMF) and refluxed under N_2 for 4 hours. The reaction mixture was cooled to room temperature and DMF and concentrated by rotary evaporation. Next, at about 60°C, 50 mL of 2M HCl was added and left stirring for 1 hour. After cooling, the solution was filtered, and the solid was washed with water and dissolved in DCM. The aqueous layer was washed 3x with DCM. The combined organic layers were dried over Na_2SO_4, filtered through glass frit, and evaporated to dryness. The product was further purified by silica gel column chromatography and precipitated with heptane to yield [(TPP)Fe^{III}(Cl)] (246 mg, 86%) (*3*).

ESI-MS (fig. S3): $[M+(K^+)]^+$ $[(TPP)Fe^{III}(Cl)(K^+)]^+$, *m/z* 742.096.

1.5. Helium Tagging Infrared Photodissociation Spectroscopy (IRPD)

Ion spectroscopic measurements were performed in our homebuilt spectrometer ISORI (Ion Spectroscopy of Organic Reaction Intermediates) using helium tagging (*4*, *5*). In a typical experiment, the intermediates/ions were generated using the VESI-MS setup by scanning a voltammogram first and then applying a constant potential where the ion abundance reached the maximum. The ions of interest ($[M]^+$) were mass-selected by a quadrupole mass filter and guided into a cold quadrupole ion trap (~3 K) by a quadrupole bender and an octopole ion guide. The ions were trapped and thermalized in collisions with helium buffer gas. Helium was injected by a piezo valve in several pulses of 0.2 ms separated by a 20 ms delay for the time of 200 ms. About 60% of the trapped $[M]^+$ ions formed helium-tagged $[M(He)_n]^+$ ($n = 1, 2$) complexes. The helium-tagged complexes ($[M(He)]^+$) were used to monitor IR photon absorption. The trapped $[M(He)]^+$ ions were irradiated by an Nd/YAG laser-pumped tunable OPO/OPA system (Laser Vision) that operated at a 10 Hz repetition rate. After the irradiation, the $[M(He)]^+$ ions were extracted from the trap, mass-analyzed by a quadrupole, and detected with a Daly-type detector working in the counting mode. The photon absorption (ν_i) was monitored as a depletion of the number of the $[M(He)]^+$ complexes. The counts of $[M(He)]^+$ complexes were measured in alternating cycles (1 Hz) with ($N(\nu_i)$) and without (N_0) the laser beam admitted to the ion trap. The infrared photodissociation (IRPD) spectra are derived as the attenuation $1-N(\nu_i)/N_0$ plotted against the infrared wavenumber.

1.6. DFT

The structure of the ions was assigned based on comparing the experimental IRPD spectra with theoretical IR spectra of different isomers and spin-isomers of the given ions. The structures of the ions were optimized with the B3LYP-D3BJ density functional theory method using the 6-311+G(2d,p) basis set. The minima were verified by calculating the Hessian matrix, which was also used to calculate harmonic IR spectra. All geometries and output files can be found in an open-data repository: https://doi.org/10.34973/27ng-ny09.

2. Experimental Results

2.1. ^1H NMR Spectrum of H_2TPP

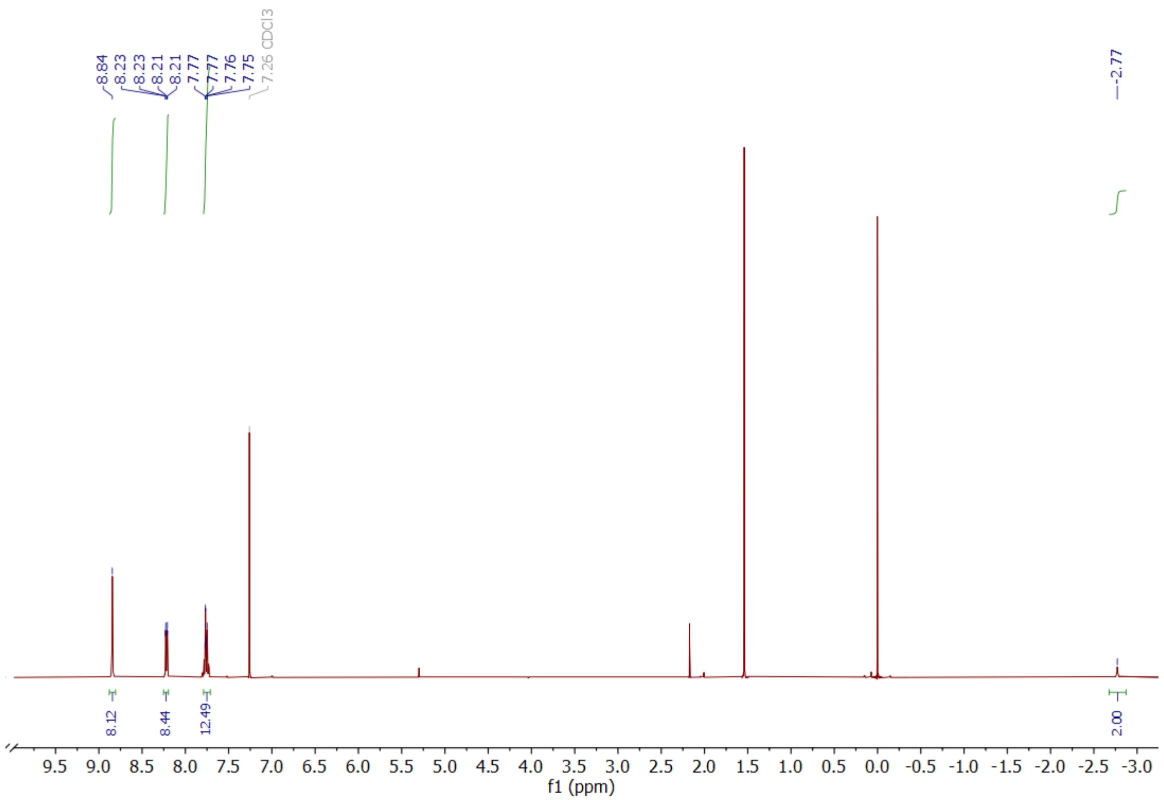

Figure S2: ^1H NMR spectrum of H_2TPP (400 MHz, $CDCl_3$)

2.2. Mass Spectrum of H_2TPP

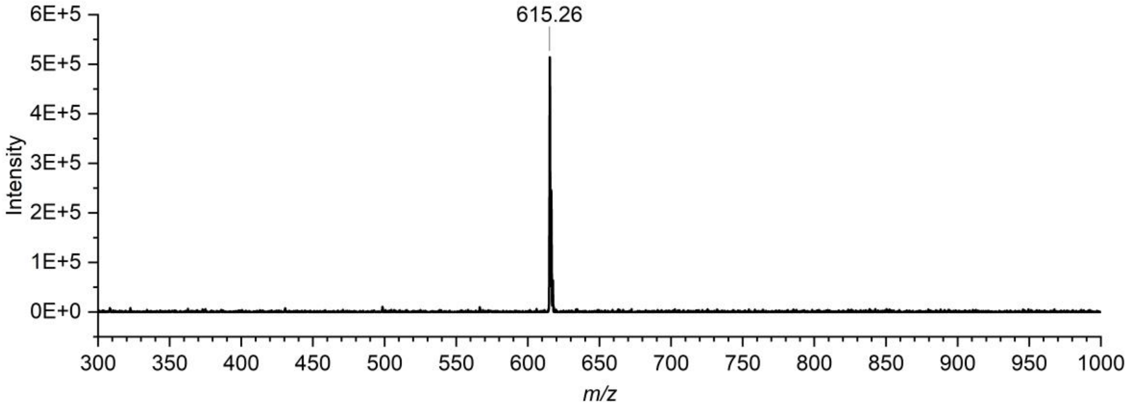

Figure S3: ESI-MS (Thermo Finnigan LCQ Deca) mass spectrum of H_2TPP $[M+H]^+$ = *m/z* 615.26

2.3. Mass Spectrum of [(TPP)Fe^{III}(Cl)]

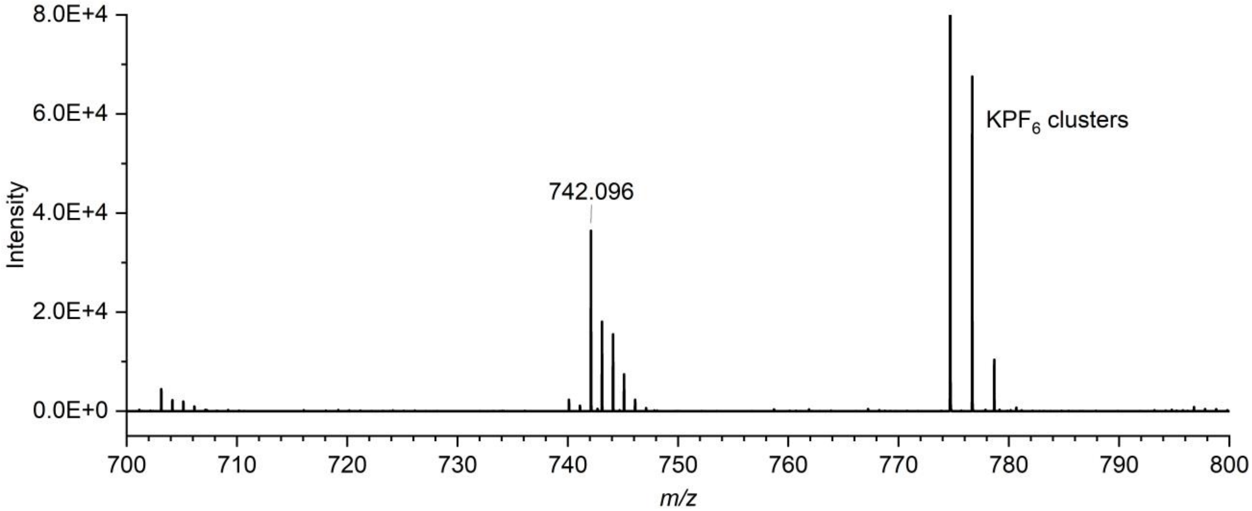

Figure S4: ESI-MS (Bruker, timsTOF) mass spectrum of [(TPP)Fe^{III}(Cl)] in the presence of KPF_6 salt, detected as [(TPP)Fe^{III}(Cl)(K^+)]$^+$, *m/z* 742.096.

2.4. Solution Resistance Measurement

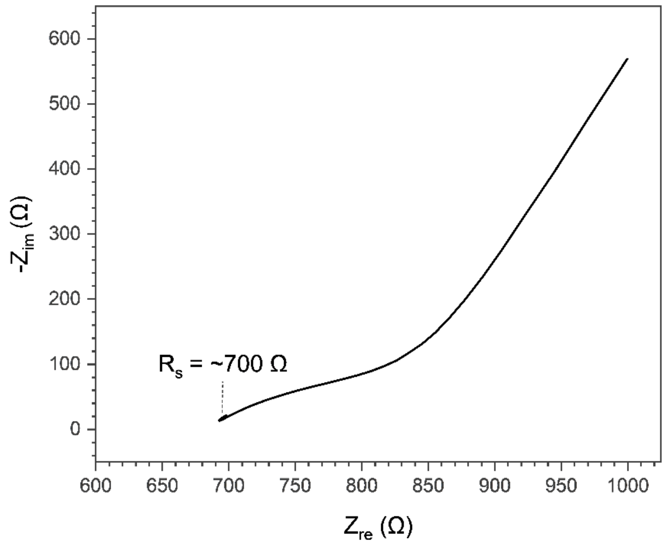

Figure S5: Solution resistance measurement: Nyquist plot for the EIS measurement at the DC potential -0.1 V vs. Pt (where there is no faradaic process) and AC amplitude of 10 mv for the frequencies 1E+05 to 10 Hz on the primary VESI-MS electrode in a solution (DCM: MeCN; 1:3) containing 2 mM KPF_6 and Fc (100 uM). The solution resistance (Rs = 700 Ω) was calculated considering the higher frequency region.

2.5. IR-Drop Correction

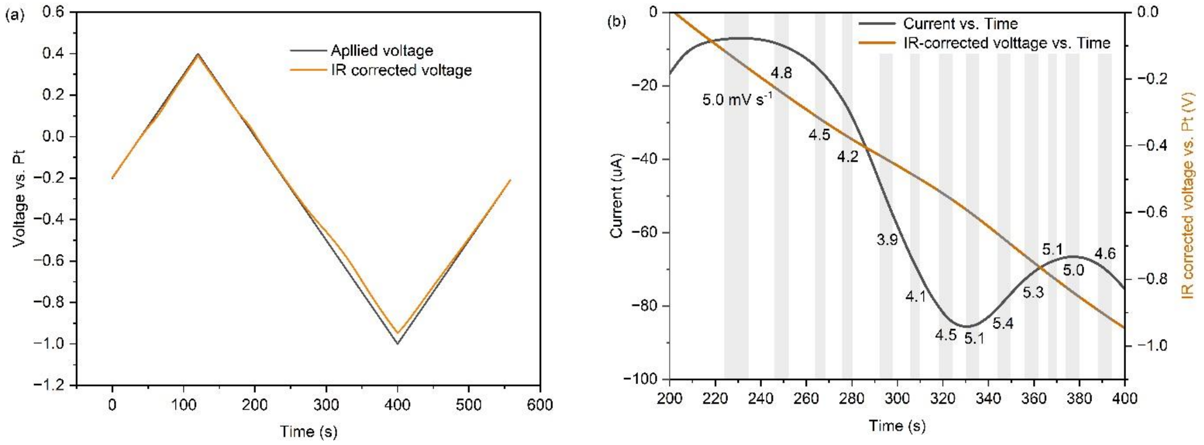

Figure S6: Post-measurement IR-drop correction of scanning potentials: VESI-MS Voltammetric scan: (a) The applied voltages compared to the IR drop corrected (post measurement correction) voltages. (b) The scan rates (slopes) at different time intervals (highlighted region) calculated from the IR-corrected scanning potential are overlayed to the voltammogram traces.

2.6. Transfer Time Determination

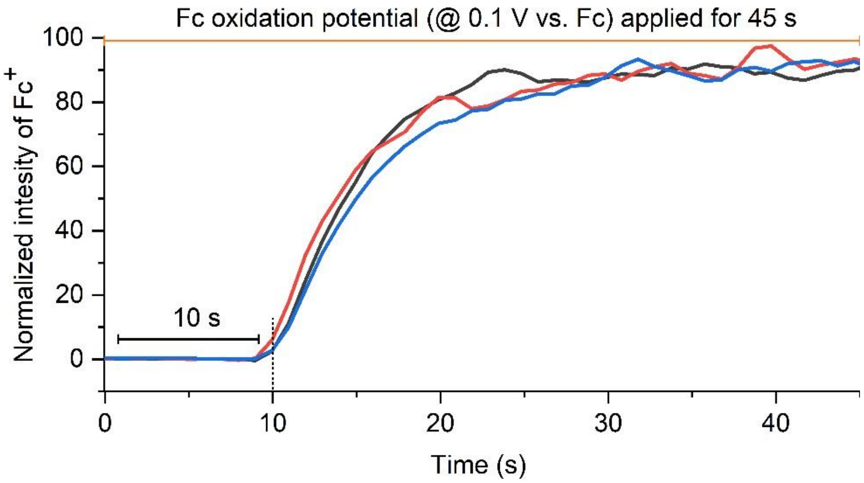

Figure S7: Determination of the transfer time: The transfer time of the species generated at the electrode to reach the mass spectrometer was determined by measuring the delay in the appearance of the Fc^+ ion signal when a pulse of the oxidation potential (0.1 V vs. Fc) was applied for 45 seconds (replicated over three measurements). The shape of the ferrocenium signal is affected by the rise of the concentration of ferrocenium in the solution infused into the ESI-MS instrument. The maximum concentration (reached after about 20 s) corresponds to the situation when the reaction is diffusion-limited, and the rate of the formation of ferrocenium is in equilibrium with the diffusion supply of ferrocene. At the beginning of the signal's rise, the rate of the ferrocenium formation is large and unlimited by the diffusion. Therefore, the signal rise is steep. Later, it slows down with the decreasing ferrocene availability at the electrode. The combination of the decreasing rate of the formation of ferrocenium and the gradual build-up of the ferrocenium concentration around the electrode is reflected in the overall shape of the ferrocenium signal.

2.7. Mass Spectra of the Intermediates Detected during VESI-MS

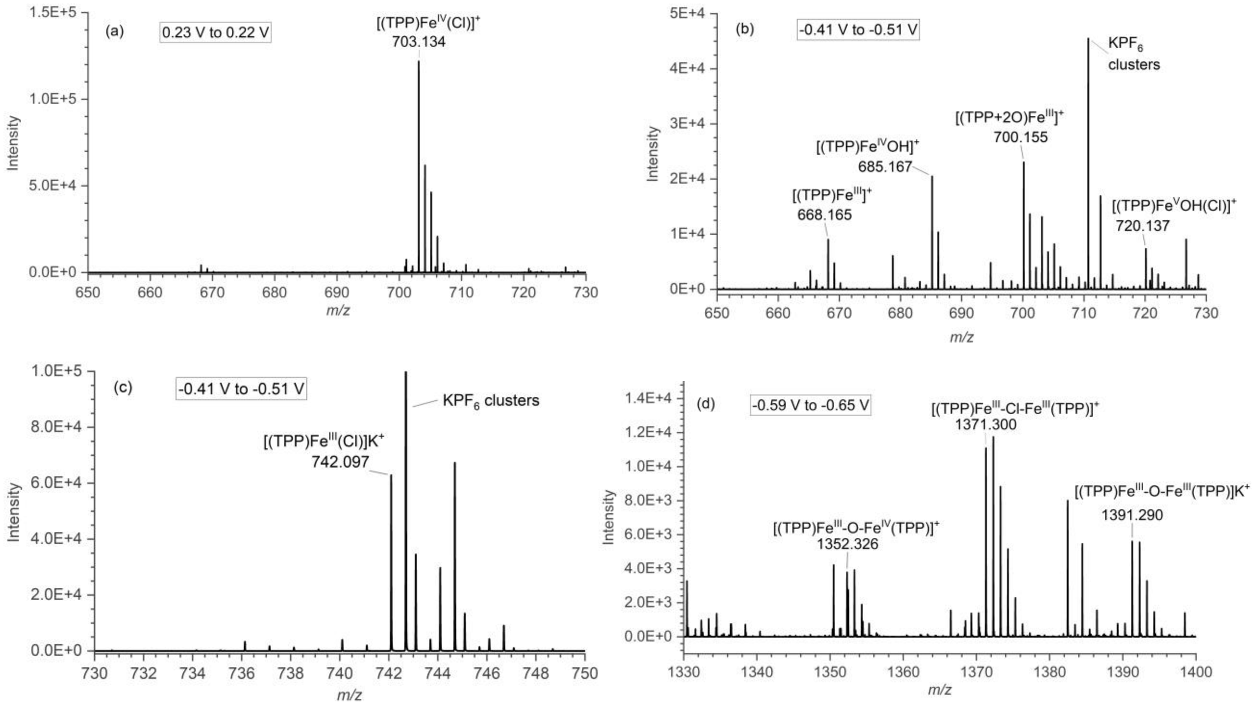

Figure S8: The mass spectra of the intermediates/ions detected during the VESI-MS analysis of electrocatalytic oxygen reduction reaction by [(TPP)Fe^{III}(Cl)] complex in the presence of 0.5 mM of TFA averaged for the indicated voltage ranges (vs. Fc/Fc^+). Experimental conditions: [(TPP)Fe^{III}(Cl)] (0.1 mM) with TFA (0.5 mM) and electrolyte KPF_6 (2 mM) in DCM-MeCN mixture (1:3), measured at a scan rate of 5 mV s^{-1} under the O_2 pressure (0.12 bar).

2.8. VESI-MS Analysis - All the Ions Detected

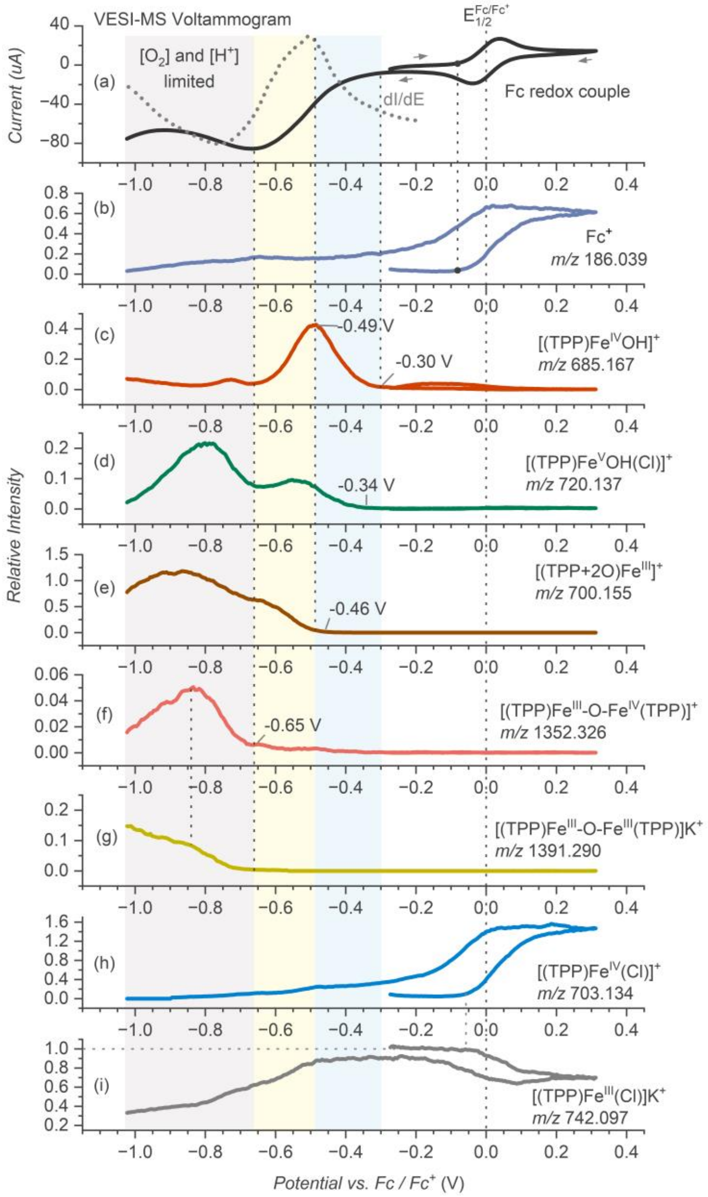

Figure S9: The VESI-MS monitoring of electrocatalytic oxygen reduction reaction by [(TPP)Fe^{III}(Cl)] complex in the presence of 0.5 mM of TFA (a) The VESI-MS voltammogram trace (solid black) and its derivative (dotted grey). The extracted ion traces of (b) Fc^+ ion (*m/z* 186.039), (c) [(TPP)Fe^{IV}OH]$^+$ (*m/z* 685.167), (d) [(TPP)Fe^{V}OH(Cl)]$^+$ (*m/z* 720.137), (e) [(TPP+2O)]Fe^{III}]$^+$ (*m/z* 700.155), (f) [(TPP)Fe^{III}-O-Fe^{IV}(TPP)]$^+$ (*m/z* 1352.326), (g) [(TPP)Fe^{III}-O-Fe^{III}(TPP)]K^+ (*m/z* 1391.290), (h) [(TPP)Fe^{IV}(Cl)]$^+$ (m/z 703.134) and (i) [(TPP)Fe^{III}(Cl)]K^+ (m/z 742.097). The ion abundances were normalized to the abundance of the parent [(TPP)Fe^{III}(Cl)] complex (detected as [(TPP)Fe^{III}(Cl)]K^+ before applying the voltage. Experimental conditions: [(TPP)Fe^{III}(Cl)] (0.1 mM) with TFA (0.5 mM) and electrolyte KPF_6 (2 mM) in DCM-MeCN mixture (1:3), measured at a scan rate of 5 mV s^{-1} under the O_2 pressure (0.12 bar).

2.9. VESI-MS Analysis at Different Concentrations of TFA

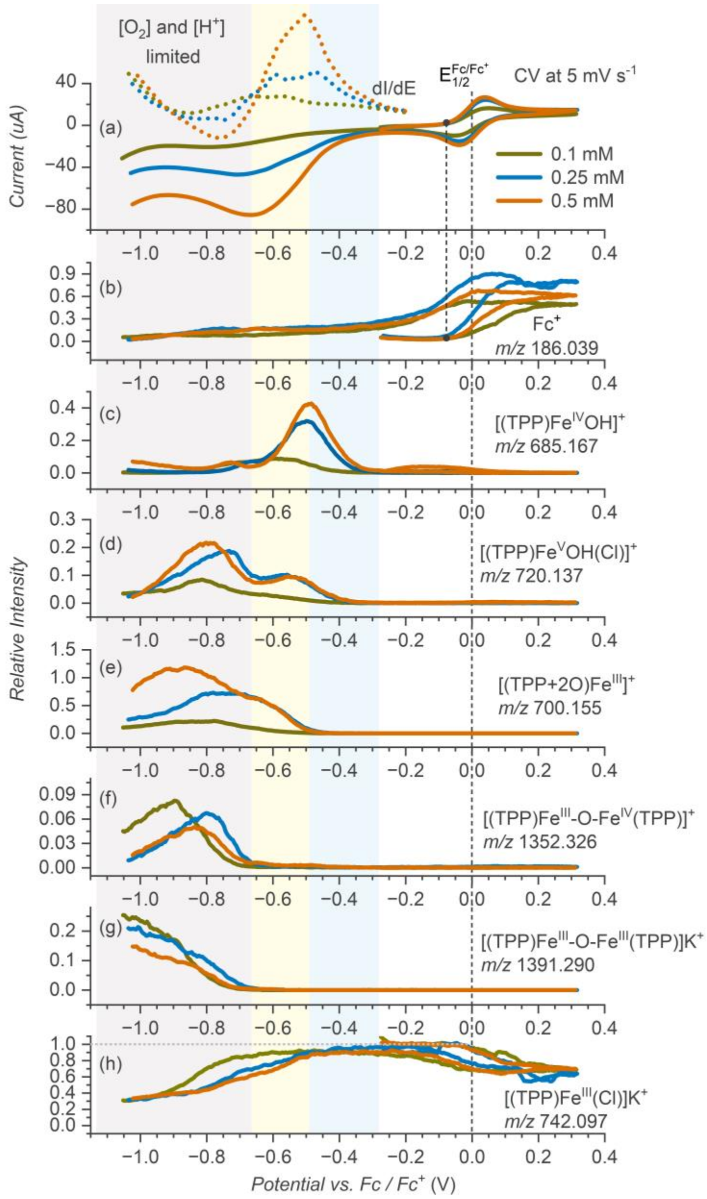

Figure S10: The VESI-MS monitoring of electrocatalytic oxygen reduction reaction by [(TPP)Fe^{III}(Cl)] complex at different concentrations of TFA (0.1mM(green), 0.25mM(blue), and 0.5mM(orange)). (a) The VESI-MS voltammogram traces (solid traces) and its derivatives (dotted traces). The extracted ion traces of (b) Fc^+ (m/z 186.039), (c) $[(TPP)Fe^{IV}OH]^+$ (m/z 685.167), (d) $[(TPP)Fe^{V}OH(Cl)]^+$ (m/z 720.137), (e) $[(TPP+2O)]Fe^{III}]^+$ (m/z 700.155), (f) $[(TPP)Fe^{III}\text{-O-}Fe^{IV}(TPP)]^+$ (m/z 1352.326), (g) $[(TPP)Fe^{III}\text{-O-}Fe^{III}(TPP)]K^+$ (m/z 1391.290), and (h) $[(TPP)Fe^{III}(Cl)]K^+$ (m/z 742.097). The ion abundances were normalized to the abundance of the parent $[(TPP)Fe^{III}(Cl)]K^+$ complex in respective measurements before applying the voltage. Experimental conditions: [(TPP)Fe^{III}(Cl)] (0.1mM) with TFA (0.1mM, 0.25mM, and 0.5mM) and electrolyte KPF_6 (2 mM) in DCM-MeCN mixture (1:3), measured at a scan rate of 5 mV s^{-1} under the O_2 pressure (0.12 bar).

2.10. VESI-MS Analysis at Different Concentrations of TFA - Correlation with Derivatives

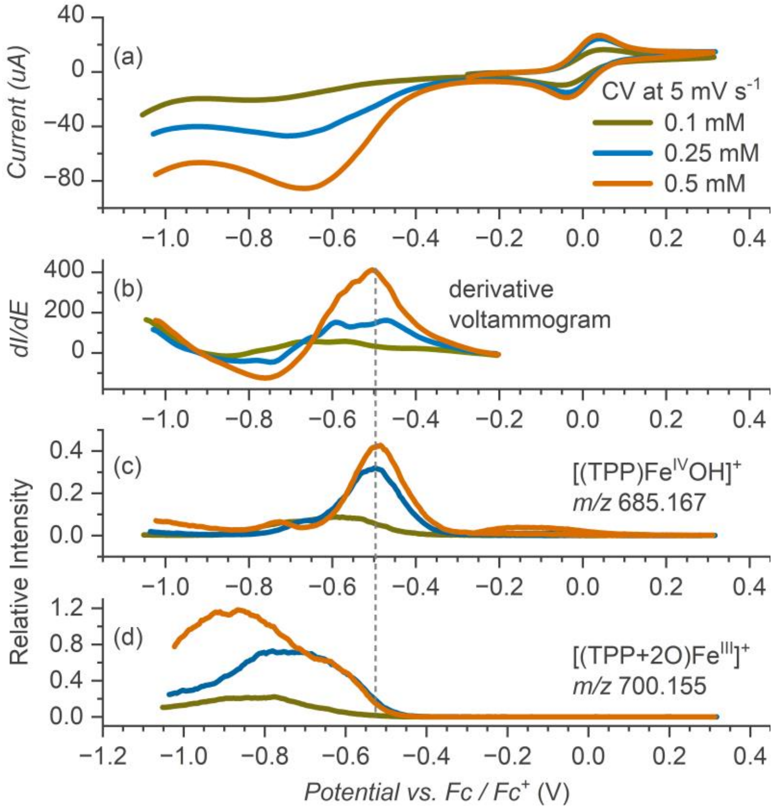

Figure S11: Correlation with the voltammogram derivatives: The VESI-MS monitoring of electrocatalytic oxygen reduction reaction by [(TPP)Fe^{III}(Cl)] complex at different concentrations of TFA (0.1 mM (green), 0.25 mM (blue), and 0.5 mM (orange)). (a) The VESI-MS voltammogram trace (b) derivatives of the VESI-MS voltammogram, the extracted ion traces of the species: (c) $[(TPP)Fe^{IV}OH]^+$ (m/z 685.167), (d) $[(TPP+2O)]Fe^{III}]^+$ (m/z 700.155). Experimental conditions: [(TPP)Fe^{III}(Cl)] (0.1mM) with TFA (0.1 mM, 0.25 mM, and 0.5 mM) and electrolyte KPF_6 (2 mM) in DCM-MeCN mixture (1:3), measured at a scan rate of 5 mV s^{-1} under the O_2 pressure (0.12 bar).

2.11. Accumulation of *m/z* 595 during chronoamperometry

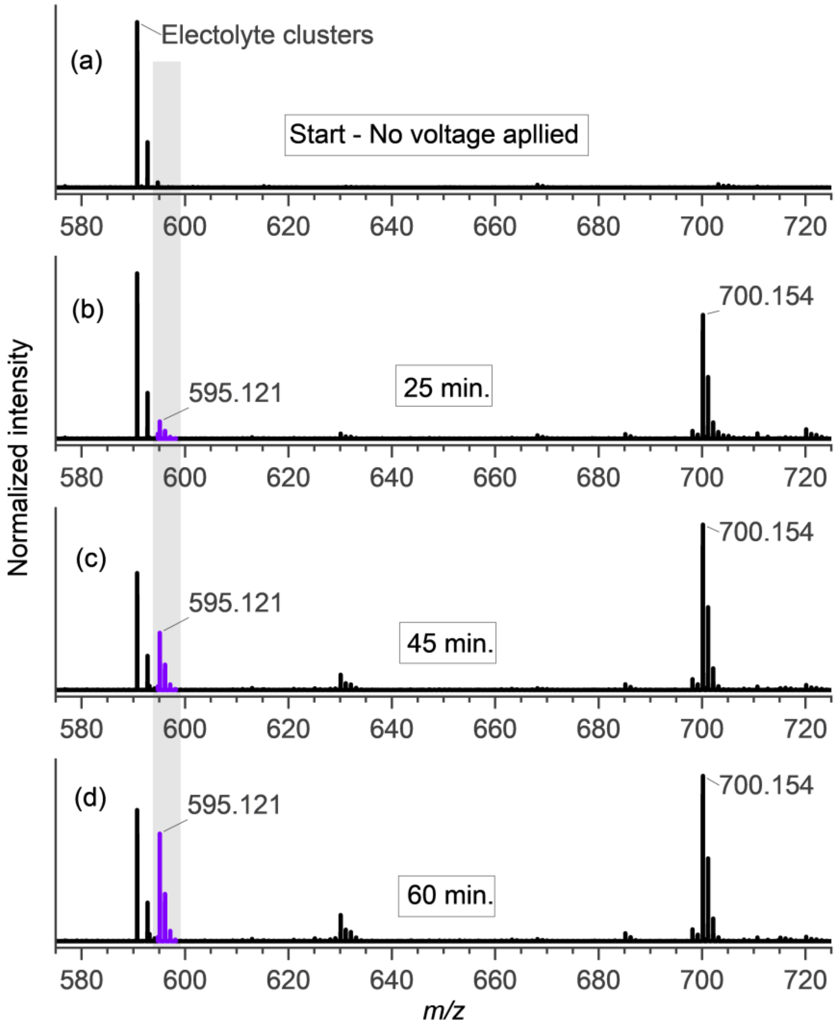

Figure S12: VESI-MS mass spectra during chronoamperometry at -0.8 V vs. Fc/Fc^+ for 1 hour: (a) before applying voltage, (b) at 25 mins, (c) at 45 mins, and (d) at 60 mins, showing the accumulation of ion m/z 595.121.

2.12. IRPD Spectrum and DFT Optimized Structures of the Intermediates

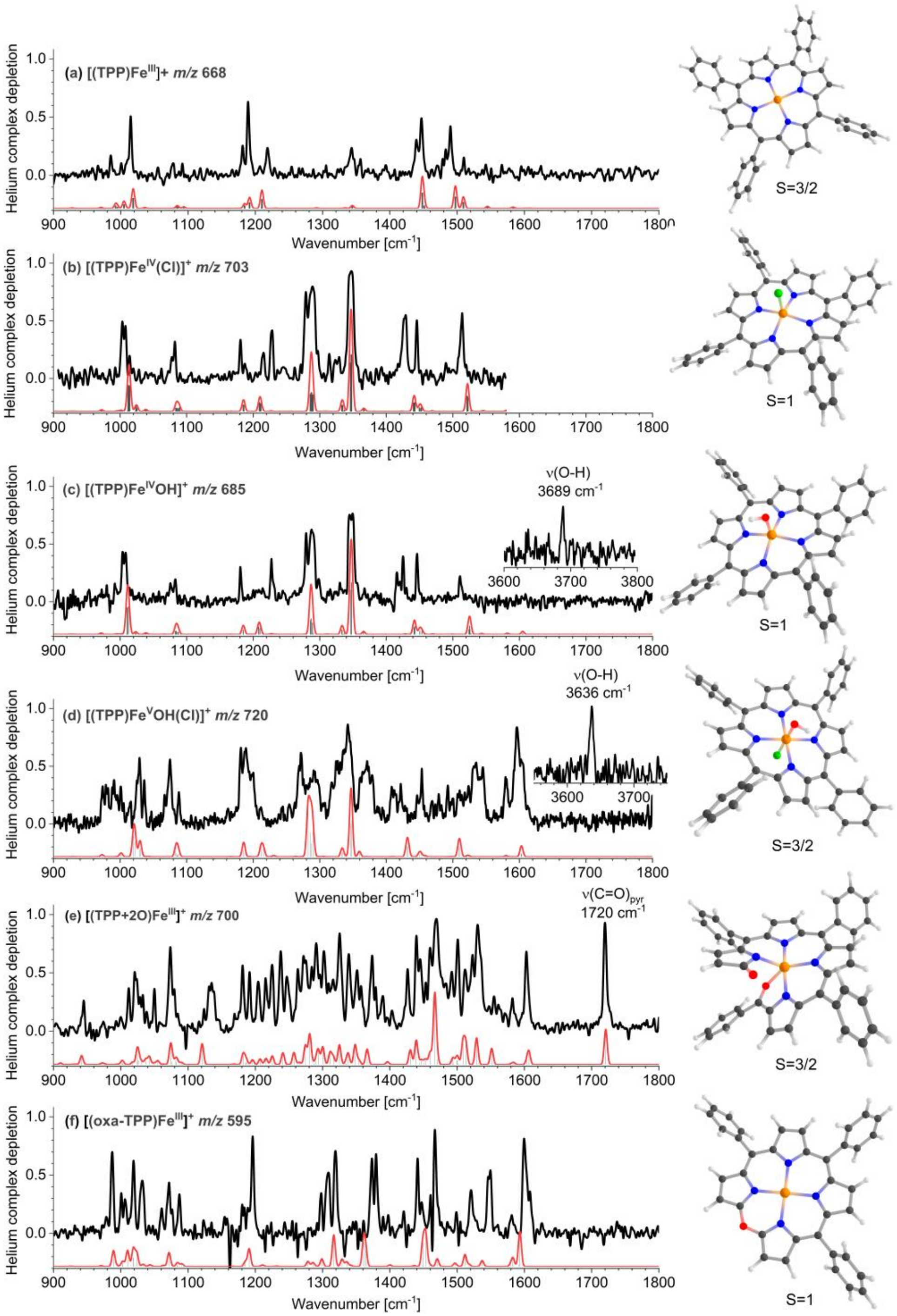

Figure S13: Helium tagging infrared photodissociation spectra of mass-selected (a) $[(TPP)Fe^{III}]^+$ (*m/z* 668.165), (b) $[(TPP)Fe^{IV}(Cl)]^+$ (*m/z* 703.134), (c) $[(TPP)Fe^{IV}OH]^+$(*m/z* 685.167), (d) $[(TPP)Fe^{V}OH(Cl)]^+$ (m/z 720.137), (e) $[(TPP+2O)]Fe^{III}]^+$ (*m/z* 700.155) and (f) $[(oxa\text{-}TPP)]Fe^{III}]^+$ (*m/z* 595.121) with DFT calculated spectra of the most stable isomers of the given ion (in red).

2.13. Isomers of $[(TPP+2O)Fe]^+$ - Comparison with IRPD Spectrum

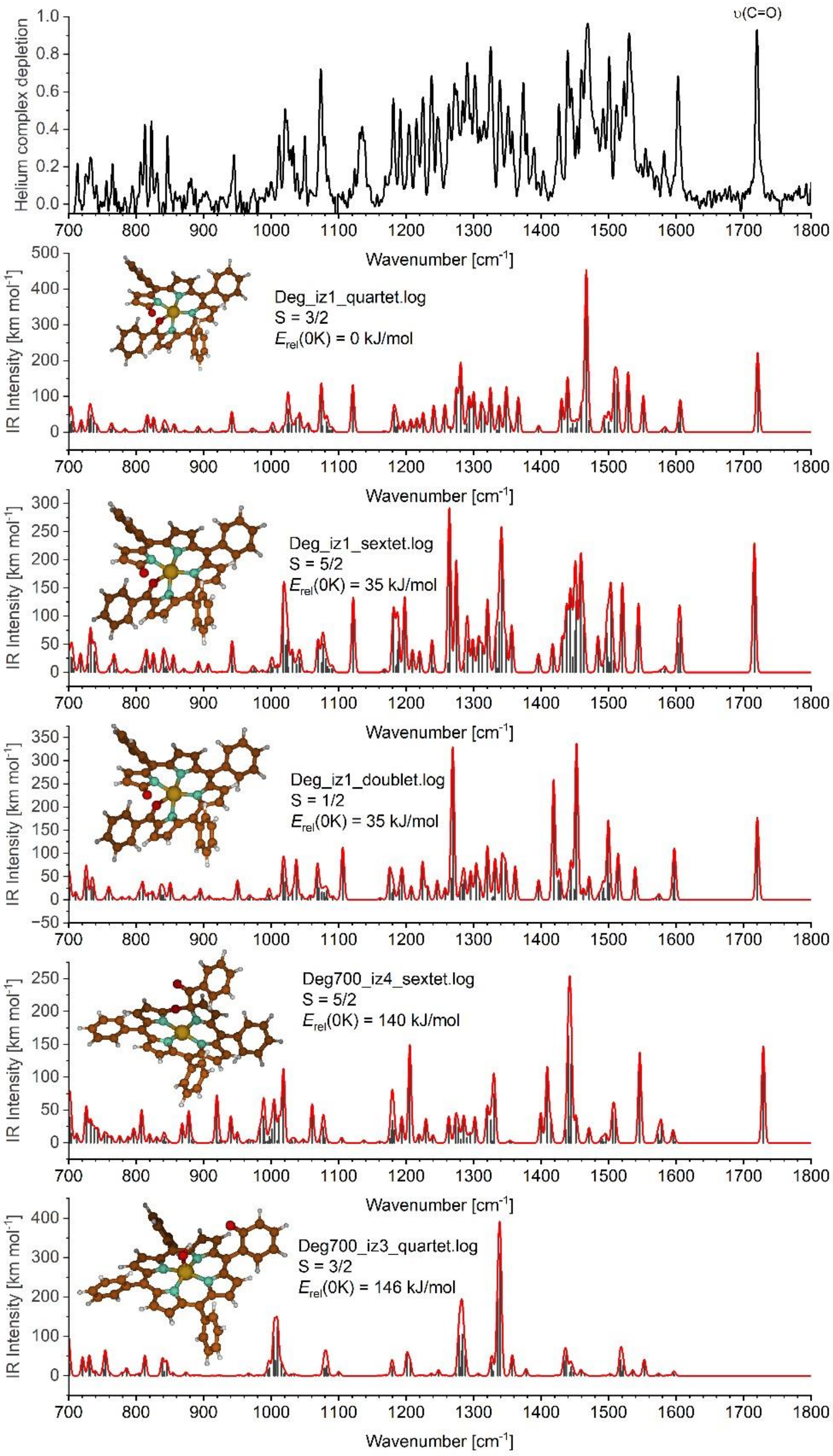

Fig. S14.

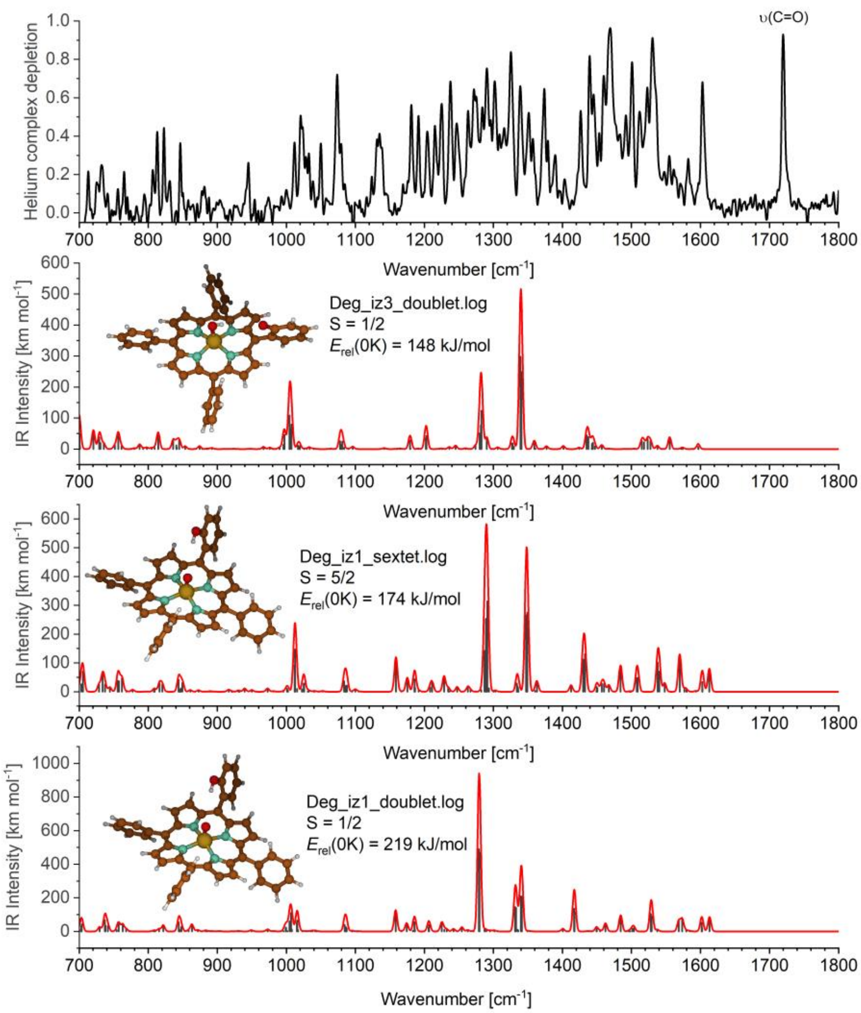

Figure S14. Continued: Comparison of the helium-tagging infrared photodissociation spectrum of ions with m/z 700.155 (top layers) with theoretical harmonic infrared spectra for different isomers of $[(TPP+2O)Fe]^+$ complexes optimized with the B3LYP-D3BJ/6-311+G (2d,p) level of density functional theory. The structures, names of the files, and relative energies at 0 K are indicated at the given spectra; the scaling factor was 0.98; the output files can be found at https://doi.org/10.34973/27ng-ny09.

2.14. Isomers of $[(TPP)Fe^{V}OH(Cl)]^{+}$ - Comparison with IRPD Spectrum

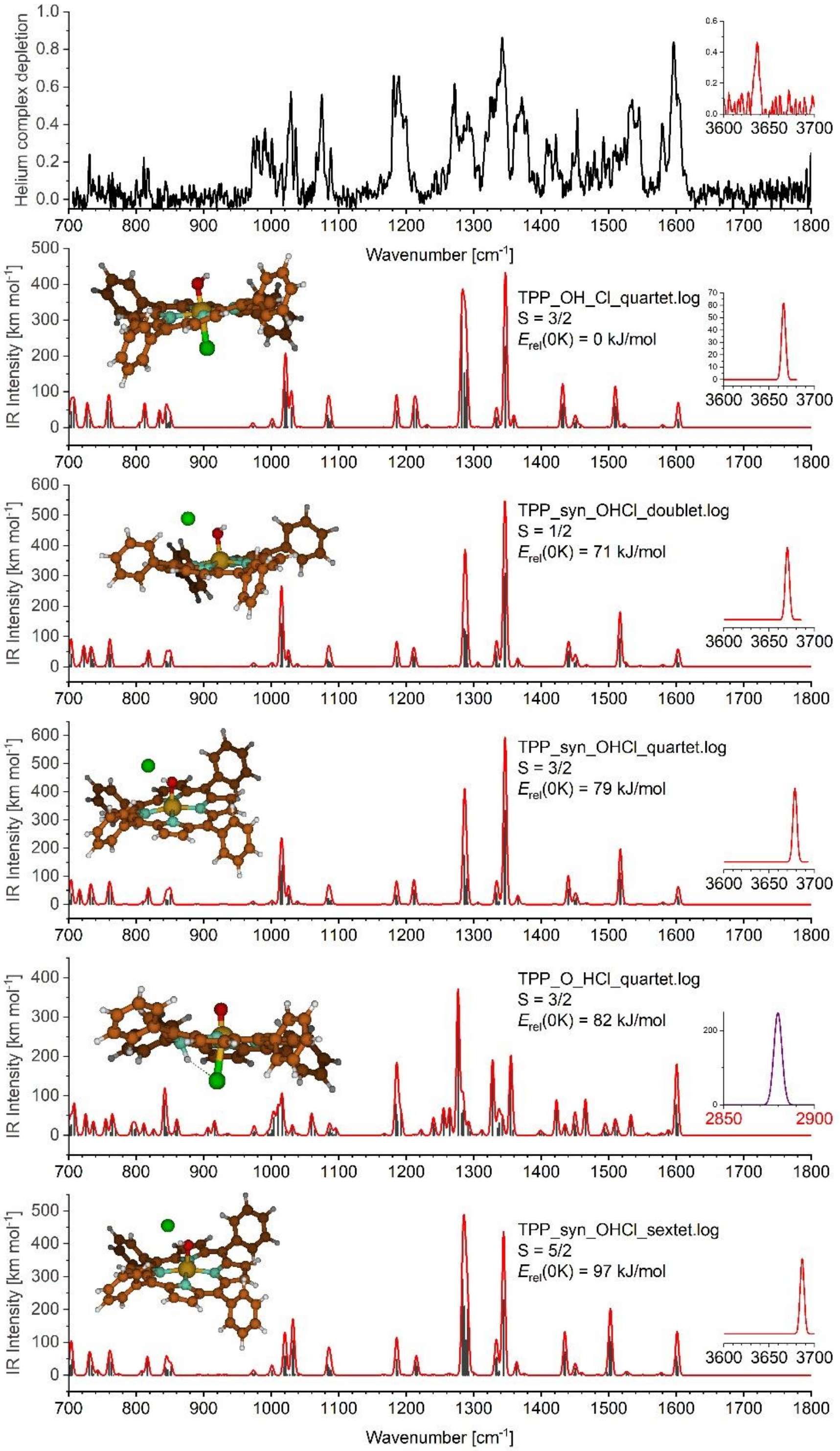

Figure S15.

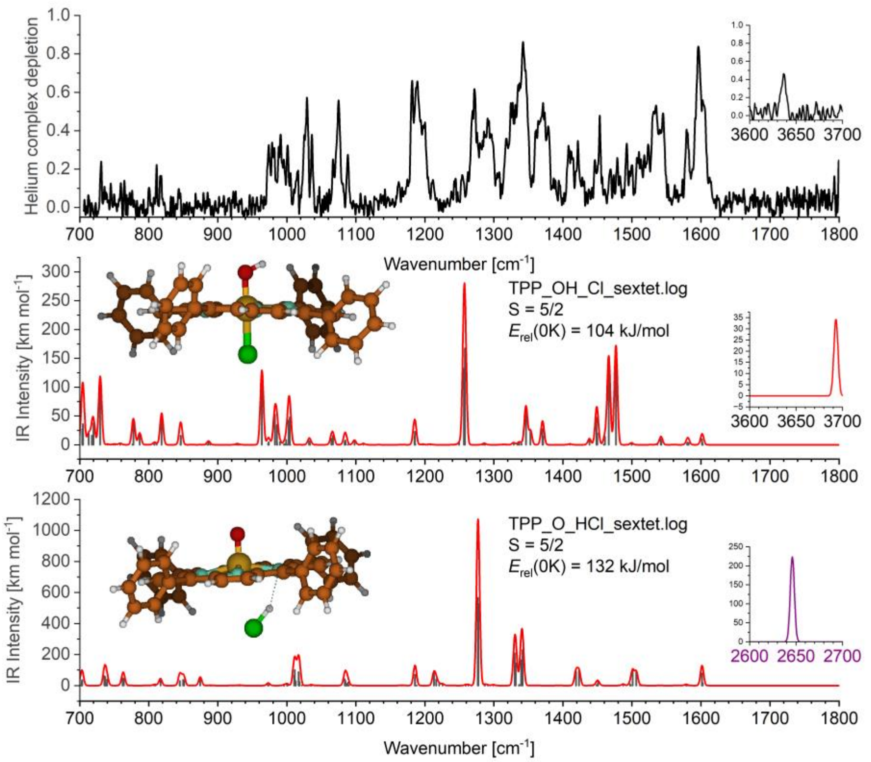

Figure S15. Continued: Comparison of the helium-tagging infrared photodissociation spectrum of the ions with *m/z* 720.137 (top layers) with theoretical harmonic infrared spectra for different isomers of $[(TPP)Fe^{V}OH(Cl)]^{+}$ complexes optimized with the B3LYP-D3BJ/6-311+G(2d,p) level of density functional theory. The structures, names of the files, and relative energies at 0 K are indicated at the given spectra; the scaling factor was 0.98 below 2500 cm^{-1} and 0.96 above 2500 cm^{-1}; the output files can be found at https://doi.org/10.34973/27ng-ny09.

2.15. Isomers of [(TPP)Fe^{IV}OH]$^+$ - Comparison with IRPD Spectrum

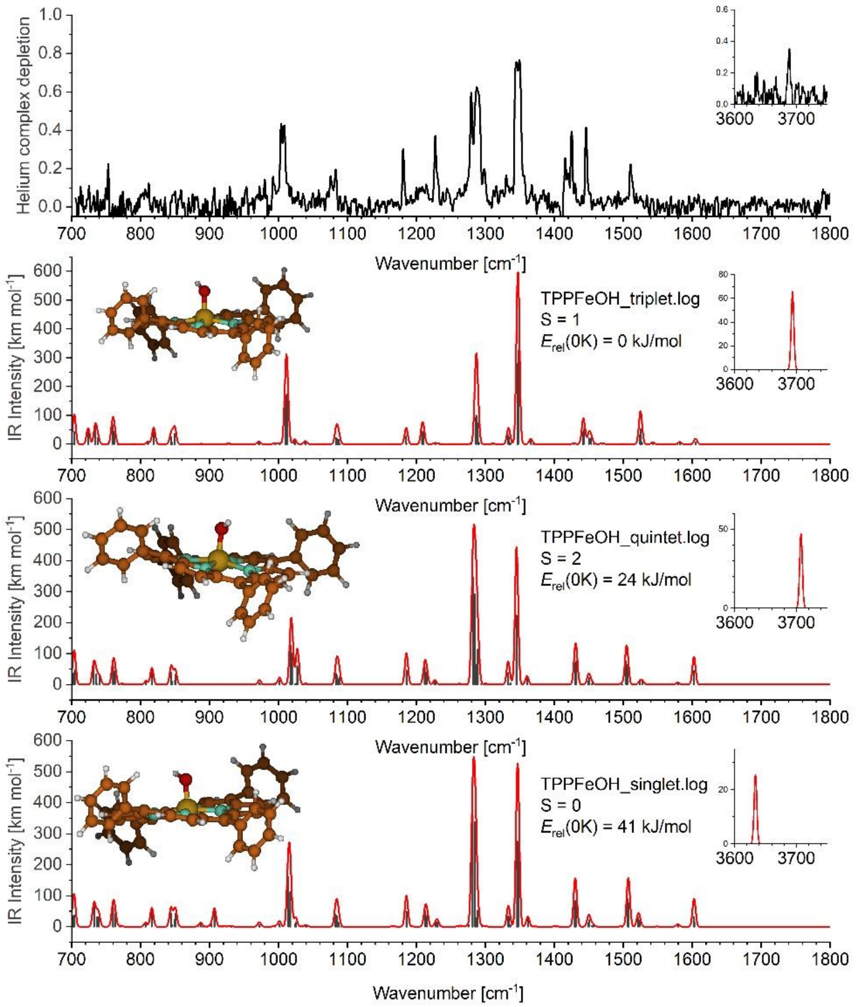

Figure S16: Comparison of the helium-tagging infrared photodissociation spectrum of the ions with *m/z* 685.167 (top layers) with theoretical harmonic infrared spectra for different spin-isomers of [(TPP)Fe^{IV}OH]$^+$ complexes optimized with the B3LYP-D3BJ/6-311+G(2d,p) level of density functional theory. The structures, names of the files, and relative energies at 0 K are indicated at the given spectra; the scaling factor was 0.98 below 2500 cm^{-1} and 0.96 above 2500 cm^{-1}; the output files can be found at https://doi.org/10.34973/27ng-ny09.

2.16. Isomers of [(oxa-TPP)Fe^{III}]$^{+}$ - Comparison with IRPD Spectrum

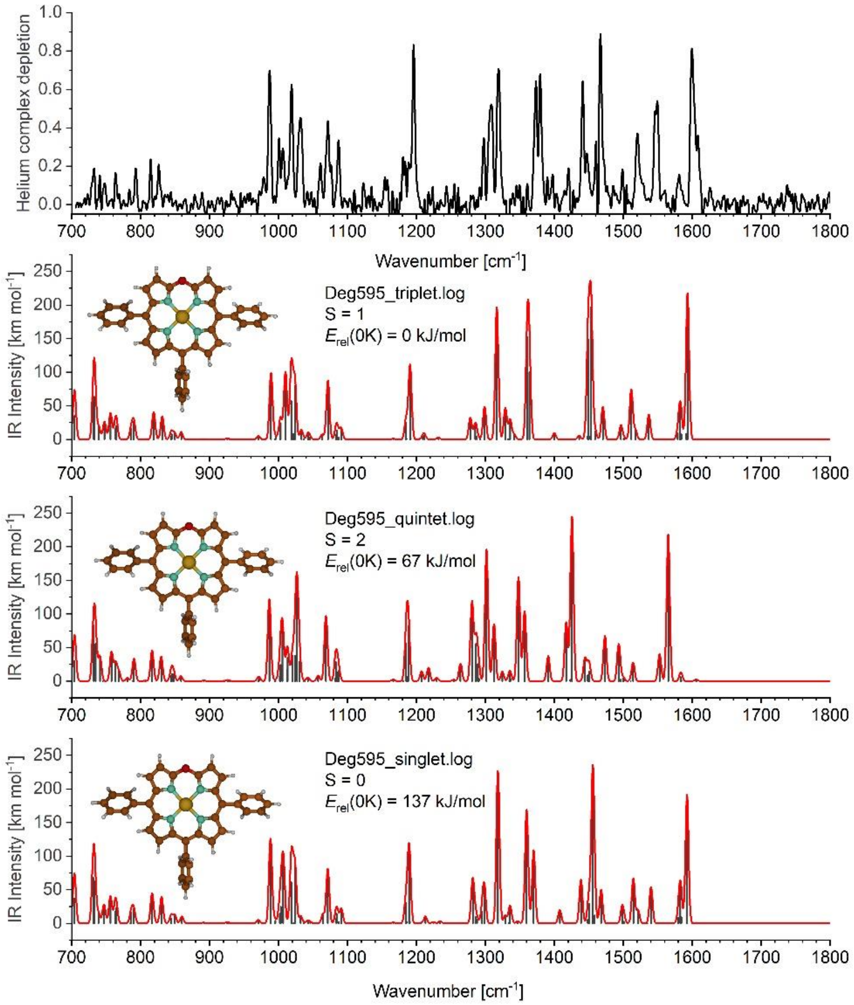

Figure S17: Comparison of the helium-tagging infrared photodissociation spectrum of the ions with *m/z* 595.121 (the top layer) with theoretical harmonic infrared spectra for different spin-isomers of [(oxa-TPP)]Fe^{III}]$^{+}$ complexes optimized with the B3LYP-D3BJ/6-311+G(2d,p) level of density functional theory. The structures, names of the files, and relative energies at 0 K are indicated at the given spectra; the scaling factor was 0.98; the output files can be found at https://doi.org/10.34973/27ng-ny09

2.17. Collision-Induced Dissociation Spectra (CID)

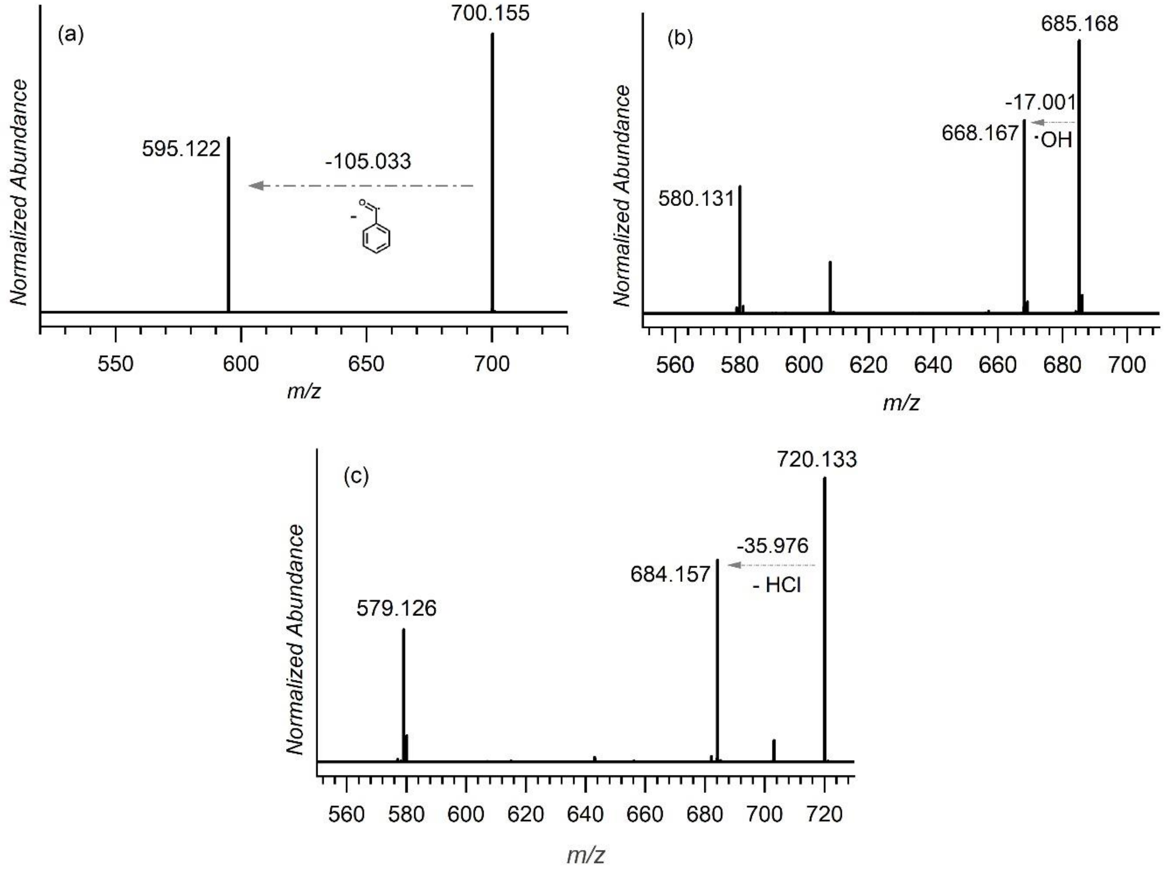

Figure S18: Collision-Induced Dissociation (CID) spectra of the ions (a) $[(TPP{+}2O)Fe^{III}]^+$ (m/z 700.155) (b) $[(TPP)Fe^{IV}OH]^+$ (m/z 685.167) (c) $[(TPP)Fe^{V}OH(Cl)]^+$ (m/z 720.137), showing the ion fragmentation pathways.

3. **References**

1. A. K. Surendran, J. Roithová, Decoding Voltammograms at the Molecular Frontier : Integration of Voltammetry and Mass Spectrometry. *Chemistry—Methods* doi.org/10.1002/cmtd.202400003 (2024).

2. S. K. Das, A. Ghosh, S. Paul Chowdhuri, N. Halder, I. Rehman, S. Sengupta, K. C. Sahoo, H. Rath, B. B. Das, Neutral Porphyrin Derivative Exerts Anticancer Activity by Targeting Cellular Topoisomerase I (Top1) and Promotes Apoptotic Cell Death without Stabilizing Top1-DNA Cleavage Complexes. *J. Med. Chem.* **61**, 804–817 (2018).

3. Z. Dou, L. Xu, Y. Zhi, Y. Zhang, H. Xia, Y. Mu, X. Liu, Metalloporphyrin-Based Hypercrosslinked Polymers Catalyze Hetero-Diels–Alder Reactions of Unactivated Aldehydes with Simple Dienes: A Fascinating Strategy for the Construction of Heterogeneous Catalysts. *Chem. – A Eur. J.* **22**, 9919–9922 (2016).

4. J. Jašík, J. Žabka, J. Roithová, D. Gerlich, Infrared spectroscopy of trapped molecular dications below 4K. *Int. J. Mass Spectrom.* **354–355**, 204–210 (2013).

5. J. Roithová, A. Gray, E. Andris, J. Jašík, D. Gerlich, Helium Tagging Infrared Photodissociation Spectroscopy of Reactive Ions. *Acc. Chem. Res.* **49**, 223–230 (2016).
